# Developing and validating ultrasound‐based machine‐learning models incorporating radiomics features to predict malignancy in adnexal masses

**DOI:** 10.1002/uog.70203

**Published:** 2026-03-30

**Authors:** F. Moro, M. Ciancia, S. di Berardino, G. Baldassari, F. Mascilini, F. Ciccarone, C. Nero, H. E. Tran, T. Pasciuto, L. Boldrini, D. Giannarelli, A. Ledger, T. Bourne, W. Froyman, D. Timmerman, L. Valentin, A. Fagotti, A. C. Testa

**Affiliations:** ^1^ UniCamillus‐International Medical University of Rome Rome Italy; ^2^ Dipartimento Scienze della Salute della Donna e del Bambino Fondazione Policlinico Universitario Agostino Gemelli IRCCS Rome Italy; ^3^ Radiomics G‐STeP Research Core Facility, Fondazione Policlinico Universitario Agostino Gemelli IRCCS Rome Italy; ^4^ Dipartimento Universitario Scienze della Vita e Sanità Pubblica, Università Cattolica del Sacro Cuore Rome Italy; ^5^ Data Collection G‐STeP Research Core Facility, Fondazione Policlinico Universitario A. Gemelli IRCCS Rome Italy; ^6^ Dipartimento di Diagnostica per Immagini, Radioterapia Oncologica ed Ematologia, Fondazione Policlinico Universitario Agostino Gemelli IRCCS Rome Italy; ^7^ Università Cattolica del Sacro Cuore Rome Italy; ^8^ Epidemiology and Biostatistics Facility, G‐STeP Generator, Fondazione Policlinico Universitario A. Gemelli IRCCS Rome Italy; ^9^ Department of Development and Regeneration, KU Leuven Leuven Belgium; ^10^ Department of Obstetrics and Gynaecology University Hospitals Leuven Leuven Belgium; ^11^ Department of Obstetrics and Gynaecology Imperial College London London UK; ^12^ Department of Obstetrics and Gynaecology Skåne University Hospital Malmö Sweden; ^13^ Department of Clinical Sciences Malmö Lund University Malmö Sweden

**Keywords:** AI, artificial intelligence, machine learning, ovarian masses, radiomics, ultrasonography

## Abstract

**Objective:**

The primary aim of this study was to develop and internally validate ultrasound‐based radiomics models to discriminate between all types of benign and malignant adnexal masses. The secondary aim was to compare the performance of the radiomics models with that of the Assessment of Different NEoplasias in the adneXa (ADNEX) model.

**Methods:**

This was a retrospective, observational, single‐center study, for which all patients with an adnexal mass that were included in the ongoing International Ovarian Tumor Analysis phase‐5 and phase‐7 studies and were examined using ultrasound between January 2012 and December 2023 at Fondazione Policlinico Universitario A. Gemelli IRCCS, Rome, Italy, were eligible for inclusion. Inclusion criteria were: adnexal mass detected by ultrasound; surgical removal of the adnexal mass within 180 days after the ultrasound examination; histological confirmation of an adnexal mass; and absence of a synchronous malignant tumor. Patients without digital ultrasound images saved in DICOM format were excluded. The patient cohort was split randomly into training and validation sets using a stratified split with a ratio of 70:30, to preserve the proportion of benign and malignant cases in the two sets. Two machine‐learning models for discriminating between benign and malignant adnexal masses were built using one image per tumor, with 5‐fold cross‐validation for hyperparameter tuning, and were tested on the validation set. The variables used in model building were patient age, serum CA 125 level and the radiomics features that differed significantly between benign and malignant tumors (determined using the Mann–Whitney *U*‐test with Benjamini–Hochberg correction) and were not redundant based on Pearson correlation analysis. Histology was the reference standard. We assessed the discriminative performance of the radiomics models using the area under the receiver‐operating‐characteristics curve (AUC) and classification performance using sensitivity and specificity at the optimal cut‐off of each model to classify the mass as malignant, as determined by Youden's index. The diagnostic performance of the developed radiomics models was compared with that of the ADNEX model (AUC, sensitivity and specificity at the 10% risk‐of‐malignancy cut‐off, which is the recommended threshold for clinical use of the ADNEX model).

**Results:**

In total, 4501 patients met the inclusion criteria. Among these, 2428 patients were excluded owing to an absence of ultrasound images or images unsuitable for radiomics analysis. Overall, a total of 2073 patients were included in the analysis, of whom 803 (38.7%) had a histologically confirmed malignant tumor. In the validation set (*n* = 622, including 254 malignancies), the clinical–radiomics model trained using the eXtreme Gradient Boosting algorithm, including age, serum CA 125 level and 14 selected radiomics features, achieved the highest performance, with an AUC of 0.89 (95% CI, 0.86–0.92), sensitivity of 0.83 (95% CI, 0.79–0.88) and specificity of 0.81 (95% CI, 0.77–0.85) at the optimal cut‐off (31% risk of malignancy, based on Youden's index). At a 10% risk‐of‐malignancy cut‐off, it had a sensitivity of 0.94 (95% CI, 0.91–0.97) and specificity of 0.48 (95% CI, 0.42–0.53). The ADNEX model had an AUC of 0.95 (95% CI, 0.93–0.97), sensitivity of 0.97 (95% CI, 0.95–0.99) and specificity of 0.72 (95% CI, 0.68–0.77) at the 10% risk‐of‐malignancy cut‐off in the validation set.

**Conclusions:**

Our results support further exploration of radiomics analysis for distinguishing between benign and malignant adnexal masses in larger study populations. Future studies should consider using multiple images per tumor and testing alternative model‐building methods, and should perform external validation to assess the generalizability of the radiomics models. © 2026 The Author(s). *Ultrasound in Obstetrics & Gynecology* published by John Wiley & Sons Ltd on behalf of International Society of Ultrasound in Obstetrics and Gynecology.

## INTRODUCTION

Tubo‐ovarian carcinoma is the eighth most common cancer in women in developed countries, with an overall 5‐year survival rate of 30–45%^1^, ^2^. An accurate preoperative diagnosis of adnexal masses is crucial for optimizing patient management and minimizing diagnostic delays^3^, ^4^, ^5^. International societies have agreed on an evidence‐based consensus statement on the preoperative diagnosis and management of patients with ovarian tumors, supporting ultrasound as the primary imaging modality for risk stratification^6^. Subjective assessment (using pattern recognition) by an expert ultrasound examiner^7^, or, alternatively, the use of the Assessment of Different NEoplasias in the adnexa (ADNEX) model^8^, is recommended to estimate the risk of malignancy and define the best patient‐management strategy. The ability of the ADNEX model to discriminate between benign and malignant masses is similar to that of an expert ultrasound examiner using pattern recognition^9^, and the model maintains its diagnostic performance when applied by a less experienced examiner^10^. The ADNEX model is the only ultrasound‐based method used in clinical practice that can calculate the probability of four different types of malignancy (borderline tumor, International Federation of Gynaecology and Obstetrics (FIGO) Stage‐I ovarian cancer, FIGO Stage‐II–IV ovarian cancer, or a metastasis to the ovary from another primary tumor). However, it relies on the ultrasound examiner to identify tumor characteristics.

Previous studies have explored the role of radiomics, a technique for extracting, analyzing and interpreting quantitative features from medical images, in classifying adnexal masses as benign or malignant^11^, ^12^, ^13^, ^14^, ^15^, ^16^, ^17^, ^18^, ^19^. However, many of these studies focused on specific patient subgroups or specific types of ovarian tumor (e.g. pregnant patients, serous ovarian tumors, tumors with specific ultrasound characteristics)^12^, ^15^, ^18^. Among the studies that included all types of ovarian tumor, the sample sizes were relatively small (*n* = 232, 241, 577, 859 and 1080, respectively)^13^, ^14^, ^16^, ^17^, ^19^. Some studies did not include any data preprocessing^12^, ^13^, ^15^, ^17^, ^18^, some were lacking independent validation^14^, ^18^, and in one study the type of machine‐learning model developed was not specified^19^.

Radiomics is subject to technical challenges (e.g. speckle noise) that require careful handling to preserve data integrity, e.g. to avoid removing informative pixel content along with the noise. Its clinical implementation demands both integration into existing workflows and validation of its added value. Despite the challenges, radiomics offers a promising solution to the key limitations of subjective ultrasound image evaluation (i.e. subjective interpretation and interobserver variability). By enabling automated, quantitative feature extraction from ultrasound images, radiomics has the potential to enhance consistency in tumor classification across different examiners.

The primary aim of this study was to develop and internally validate ultrasound‐based radiomics models to discriminate between all types of benign and malignant adnexal masses. The secondary aim was to compare the performance of the radiomics models with that of the ADNEX model.

## METHODS

### Study design

This was a retrospective, observational, single‐center study conducted using data from the ongoing International Ovarian Tumor Analysis (IOTA) phase‐5^9^ and phase‐7 (ClinicalTrials.gov no.: NCT02847832) prospective observational studies (Appendix S1), collected at the referral oncology center Fondazione Policlinico Universitario A. Gemelli IRCCS, Rome, Italy, between January 2012 and December 2023.

Inclusion criteria were: adnexal mass detected by ultrasound; surgical removal of the adnexal mass within 180 days after the ultrasound examination; histological confirmation of an adnexal mass; and absence of synchronous malignant tumor. Patients without digital ultrasound images saved in DICOM format were excluded. The protocol was approved by the regional ethical committee responsible for Fondazione Policlinico Universitario A. Gemelli IRCCS (Lazio Region Ethics Committee 3; Prot. N. 0001675/24). The Transparent Reporting of a multivariable prediction model for Individual Prognosis Or Diagnosis + Artificial Intelligence reporting guidelines were followed^20^.

Information on clinical characteristics (age, parity, menopausal status, serum CA 125 level, presence or absence of symptoms) and ultrasound variables (unilateral or bilateral tumor, side and origin of tumor, maximum diameter of tumor, maximum diameter of the largest solid component of the tumor, number of cyst locules, echogenicity of cyst fluid, presence of acoustic shadows, number of papillary projections, presence of ascites, color score)^21^ was retrieved from Clinical Data Miner^22^, an electronic case‐reporting system used in both the IOTA‐5 and IOTA‐7 studies. In the case of bilateral masses, the mass with the ultrasound morphology most indicative of malignancy was included in the analysis; if the masses had similar sonographic morphology, the largest mass or the mass best assessable using ultrasound was included. Information on the final histological diagnosis and FIGO stage of each mass was retrieved from the IOTA database, with histology used as the reference standard.

Patients were examined using different types of ultrasound machines (Appendix S2, Table S1). Ultrasound examinations were carried out using standardized IOTA examination and measurement techniques, and IOTA terminology was used to describe the ultrasound findings^21^. All examiners performing the ultrasound scans were experienced and fulfilled the criteria of a European Federation of Ultrasound Societies in Medicine and Biology (EFSUMB) Level‐II or ‐III examiner^23^. Subjective assessment (pattern recognition) of the ultrasound images by the ultrasound examiner was used to guide patient management.

### Image segmentation and feature extraction

Ultrasound images from each patient, saved in DICOM format, were retrieved from external hard disks or the Picture Archiving and Communication System. For an image to be used in radiomics analysis, it must be grayscale without calipers, text or a color‐Doppler box. If more than one image suitable for radiomics analysis was available for a patient, the image with the best quality (i.e. well‐defined tumor margins, well‐visualized solid component) that was most representative of the tumor according to an EFSUMB level‐II examiner (M.C.) was selected for analysis.

The region of interest (ROI) of each image was manually segmented by five EFSUMB Level‐I or ‐II examiners using the software Aliza version 1.48 (Mikhail Isakov, Bonn, Germany). All five examiners received training prior to performing manual segmentation and were provided with at least one reference ultrasound image with calipers indicating the borders of the adnexal mass to guide the segmentation process. A single EFSUMB level‐II examiner (M.C.) reviewed all segmented images and resegmented them if the segmentation was not accurate. All ultrasound images were preprocessed using a Wiener filter with kernel size 3 × 3 to reduce the speckle noise^24^. Image intensities were normalized using *Z*‐scores to standardize images across different patients. Normalization was performed for each ROI individually by first subtracting the mean intensity value of the ROI from the intensity of each pixel. This difference was then divided by the SD of the intensity value, calculated within the same ROI. *Z*‐score normalization was applied within each ROI to standardize pixel intensities, allowing for relative contrast comparisons and reducing scanner‐related and interpatient variability. This normalization may introduce bias, particularly for intensity‐based statistical features, as scaling by the mean and SD may attenuate absolute intensity differences that encode biologically meaningful heterogeneity between tumors. For textural features that assess local patterns and distributions, the bias introduced by *Z*‐score normalization is probably minimal.

Radiomics features were extracted using the MODDICOM platform^25^. This is an open‐source, in‐house software developed by Knowledge Based Oncology (KBO) Labs (Rome, Italy) for quantitative image analysis that is fully compliant with the Image Biomarker Standardisation Initiative recommendations^26^. The extracted features can be subcategorized into intensity‐based statistical features and textural features (Appendix S3)^27^.

### Model development

Sample‐size calculation was not performed prior to the study. The cohort of patients was split randomly into training and hold‐out validation sets using a stratified split with a ratio of 70:30, to ensure the same proportion of benign and malignant (including borderline, early stage, advanced and metastatic ovarian tumors) masses in the two sets. The 70:30 training/validation split was chosen because of the large sample size^28^; this ratio ensured sufficient data for effective model training while still allowing for accurate evaluation of unseen cases. According to Collins *et al*.^29^, a validation set should include at least 100 events. Using the 70:30 split, our validation set included over 250 malignant masses (events).

For the radiomics analysis, the Mann–Whitney *U*‐test was used to identify radiomics features that differed significantly between benign and malignant tumors in the training set. Two‐tailed *P* < 0.05 was considered statistically significant. Subsequently, these *P*‐values were adjusted for multiple testing using the Benjamini–Hochberg procedure, with the significance level set at 0.05. For the development of the machine‐learning models, the correlation between radiomics features that exhibited significant differences between benign and malignant tumors (determined using Mann–Whitney *U*‐test with Benjamini–Hochberg correction) was investigated using the Pearson correlation coefficient. To avoid multicollinearity, when two or more features were highly correlated (correlation coefficient > 0.6^30^), we included in the model the feature with the lowest *P*‐value based on the Mann–Whitney *U*‐test. To bring the values of the selected radiomics features to a common scale, they were normalized using *Z*‐score normalization scaling.

Additional feature‐selection methods (minimum redundancy maximum relevance (MRMR) and Recursive Feature Elimination (RFE)) were subsequently applied in the case of overfitting^31^, ^32^. Principal Component Analysis (PCA) was performed in the training set to investigate the impact of the ultrasound system manufacturer on the selected radiomics features.

Two machine‐learning models were created using the training set and tested on the hold‐out validation set regarding their ability to discriminate between benign and malignant adnexal masses: a radiomics model including only radiomics features (radiomics‐only model), and a radiomics model including age, serum CA 125 level and radiomics features (clinical–radiomics model). The events‐per‐variable (EPV) ratio was calculated to ensure that the number of selected radiomics features was appropriate in relation to the number of outcome events. The ratio was calculated as the number of malignant cases in the validation cohort divided by the number of radiomics features, following the recommendations of Peduzzi *et al*.^33^.

Four machine‐learning classifiers were tested for building the radiomics‐only model, including logistic regression with penalties, random forest, eXtreme Gradient Boosting (XGBoost) and support vector machine, and the best performing model was chosen. For the clinical–radiomics model, only random forest and XGBoost were trained, as these algorithms natively handle missing values (e.g. serum CA 125 level), thereby eliminating the need for data imputation. To identify the optimal set of hyperparameters (parameters characteristic of the machine‐learning model that modify and guide the learning process), fine‐tuning was performed with a randomized grid search using a 5‐fold cross‐validation over the training set. To assess which clinical and radiomics features contributed most to the classification, a feature importance analysis was performed on the clinical–radiomics and radiomics‐only models in the training set. Importance was calculated based on the ‘gain’, which represents the average improvement in model accuracy when a feature is used to split the data in a decision tree.

We assessed which features contributed most to overfitting of the radiomics‐only model using SHapley Additive exPlanations (SHAP) values^34^. We calculated the mean absolute SHAP value of each feature in the training and validation sets. Features with a high SHAP value in the training set but a lower SHAP value in the validation set were judged to be possibly overfitted. We defined an ‘Overfit_Score’ as the difference in the SHAP value of a feature between the training and validation sets.

Information on the exact software versions, required dependencies, preprocessing pipeline and feature extraction procedures is provided in the GitHub repository: https://github.com/Radiomics‐gstep/RADIANTES. The models can be imported using Python (version 3.11.7) after preprocessing the DICOM ultrasound images and extracting and normalizing the selected radiomics features. All these steps can be performed using the tools and scripts available in the repository.

### Validation of model performance

The performance of the models was assessed using receiver‐operating‐characteristics (ROC) curves, and their discriminative ability to distinguish between benign and malignant masses was expressed as the area under the ROC curve (AUC). We assessed the performance of each model to classify a mass as malignant, calculating sensitivity, specificity, positive predictive value, negative predictive value, positive likelihood ratio and negative likelihood ratio at a 10% risk‐of‐malignancy cut‐off (the recommended cut‐off for clinical use of the ADNEX model^6^) with their 95% CIs. We also assessed classification performance when using the optimal cut‐off of each radiomics model to classify the mass as malignant (i.e. the cut‐off providing the largest number of correct classifications according to Youden's index^35^). We used normal approximation to estimate the 95% CIs for all metrics except AUC, for which we used bootstrapping. The statistical significance of any difference in AUC between the two radiomics models and the ADNEX model was calculated using the DeLong method^36^.

We evaluated the calibration of the radiomics models and the ADNEX model by calculating the calibration intercept and slope using a logistic recalibration model. Calibration curves were generated using a non‐parametric approach. The calibration intercept indicates whether predicted risks are, on average, systematically overestimated (intercept < 0) or underestimated (intercept > 0). The calibration slope reveals whether risks are overly extreme (i.e. low risks are underestimated and high risks are overestimated (slope < 1)) or too moderate (i.e. low risks are overestimated and high risks are underestimated (slope > 1))^37^.

We evaluated clinical utility using decision‐curve analysis with risk thresholds (exchange rates) ranging from 1% to 50%, to determine which patients to refer to an oncology center.

The diagnostic performance of the two radiomics models was compared with that of the ADNEX model and with subjective assessment by the original ultrasound examiner. We applied the ADNEX model to our training and validation sets and computed the risk of malignancy using the ADNEX formulae (Appendix S4). The risk of malignancy estimated using the ADNEX model was calculated incorporating serum CA 125 level when it was available at the time of inclusion.

### Statistical analysis

Categorical variables are presented as *n* (%) while continuous variables are presented as median (interquartile range (IQR)), reported separately for the training and validation sets. Statistical differences between benign and malignant tumors were assessed using the chi‐square test for categorical variables (or Fisher's exact test, when appropriate) and the Mann–Whitney *U*‐test for continuous variables. The Kruskal–Wallis test was used to evaluate differences between subtypes of ovarian malignancies. Statistical analysis and modeling were performed using RStudio (Posit PBC, Boston, MA, USA) with R version 4.2.2 (R Foundation for Statistical Computing, Vienna, Austria) and Visual Studio Code (Microsoft, Redmond, WA, USA) with Python 3.11.7 (Python Software Foundation, Wilmington, DE, USA).

## RESULTS

### Clinical, ultrasound and histopathological features

**Figure 1:**
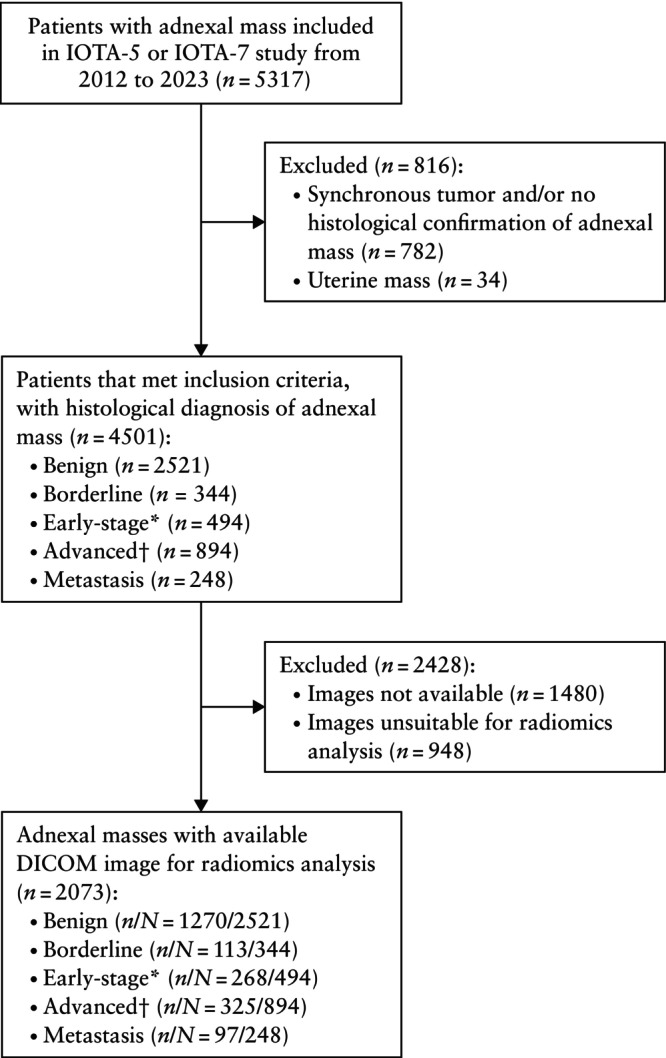
Flowchart showing inclusion of women with adnexal mass in study cohort. *International Federation of Gynecology and Obstetrics (FIGO) Stage I–II. †FIGO Stage III–IV. IOTA, International Ovarian Tumor Analysis.

A total of 5317 patients were identified from the IOTA‐5 and IOTA‐7 databases. Among these, 4501 patients met the inclusion criteria. After excluding patients with no available images (*n* = 1480) or images that were unsuitable for radiomics analysis (*n* = 948), 2073 patients were included in the analysis (Figure 1), of whom 803 (38.7%) had a histologically confirmed malignant tumor. The clinical and ultrasound characteristics of patients included and excluded from the final analysis are described in Table S2. The proportion of histologically confirmed malignant tumors and advanced malignancy was higher among the excluded patients *vs* included patients (malignant: 48.5% *vs* 38.7%; advanced malignancy: 71.6% *vs* 54.8%). Although there were significant differences in the ultrasound appearance of tumors between included and excluded patients, these differences were small, the most significant difference being a greater proportion of tumors without acoustic shadowing in the excluded group, as assessed on baseline ultrasound examination. In total, 1293/2073 (62.4%) included patients were examined in the years 2021, 2022 and 2023 *vs* 539/2428 (22.2%) of those excluded (Table S3). The number of available ultrasound images per included patient is presented in Table S4, showing that 1533/2073 (74.0%) patients had only one image suitable for radiomics analysis. Overall, 1270/2073 (61.3%) included patients had a benign tumor and 803/2073 (38.7%) had a malignant tumor, of which 113/803 (14.1%) were borderline tumors, 268/803 (33.4%) were early‐stage (FIGO Stage I–II) invasive tumors, 325/803 (40.5%) were advanced (FIGO Stage III–IV) invasive tumors and 97/803 (12.1%) were metastases from another primary tumor. Information on serum CA 125 level was available for 1552/2073 included patients (1092/1451 (75.3%) in the training set and 460/622 (74.0%) in the validation set) and was unavailable for 521/2073 patients (359/1451 (24.7%) in the training set and 162/622 (26.0%) in the validation set).

Among 2073 included patients, 1451 were grouped into the training set and 622 into the validation set. Clinical and ultrasound characteristics are described separately for the training and validation sets in Tables S5 and S6, respectively. No significant clinical differences were found between the training and validation sets. The median age was 51 years in both sets, the proportion of postmenopausal patients was identical (51.3%) and a similar proportion of patients were symptomatic (41.6% in the training set *vs* 41.8% in the validation set). There were no significant differences in the ultrasound characteristics of the tumors between the two subsets. On baseline ultrasound examination, the median largest tumor diameter was 66 (IQR, 44–101) mm in the training set and 65 (IQR, 44–99) mm in the validation set, and the median maximum diameter of the largest solid component was 16 (IQR, 0–54) mm in the training set and 18 (IQR, 0–59) mm in the validation set. Tumors were multilocular with more than 10 cyst locules in 13.6% of patients in the training set and 13.3% in the validation set. Papillary projections were present in 22.2% of tumors in both the training and validation sets. Acoustic shadows were identified in 22.4% of tumors in the training set and 21.7% in the validation set, and ascites was detected in 9.7% and 10.3% of patients, respectively.

The histological diagnosis and FIGO stage of tumors are shown in Tables S7 and S8, respectively. The distribution of early‐stage (FIGO Stage I–II) and advanced (FIGO Stage III–IV) ovarian tumors was similar in the two sets; early‐stage malignant tumors accounted for 12.1% of tumors in the training set and 14.8% in the validation set, while advanced tumors represented 15.5% and 16.1% of tumors in the training and validation sets, respectively. The most frequent benign histological diagnosis was teratoma in the training set (12.7% of all tumors) and endometrioma in the validation set (11.3% of all tumors), and the most frequent borderline ovarian tumor was the serous histotype in both the training and validation sets. Among early‐stage tumors, the most frequent histotype was serous ovarian cancer, accounting for 4.3% of all tumors in the training set and 5.0% of all tumors in the validation set, followed by endometrioid ovarian cancer (3.0% in the training set and 4.7% in the validation set). The serous histotype was also the most common among advanced ovarian cancers, accounting for 13.4% of all tumors in the training set and 14.3% of all tumors in the validation set. Most metastatic ovarian cancers were of gastrointestinal origin in both the training and validation sets (2.5% and 2.6% of all tumors, respectively).

### Model development

A total of 74 radiomics features were extracted from each ROI. Of these, 56 features were found to be significantly different between benign and malignant tumors based on the Mann–Whitney *U*‐test adjusted using the Benjamini–Hochberg procedure. After removing correlated variables using Pearson correlation analysis, 14 representative features were included as input variables in both radiomics models (Appendix S5). In the clinical–radiomics model, data on serum CA 125 level and age were also incorporated, in addition to the 14 features. The best performance of both the radiomics‐only model and the clinical–radiomics model in the training set was provided by XGBoost, which yielded the highest AUC. PCA revealed that the distributions of the 14 selected radiomics features overlapped across different types of ultrasound machine (Figure S1), demonstrating that the ultrasound machine manufacturer had no significant impact on the extraction of these features.

The EPV ratio for the clinical–radiomics model was 15.88, calculated as the ratio of 254 malignant cases in the validation cohort to 16 predictors (14 radiomics features, age and serum CA 125 level). This value aligns with the acceptable range (EPV > 10)^33^ and approaches the optimal number of events (200) recommended by Collins *et al*.^29^, indicating an adequate sample size for reliable estimation of model performance and a reduced risk of overfitting.

Feature importance analysis revealed that CA 125 was the most impactful feature in the clinical–radiomics model, with an importance value of 0.19, followed by the radiomics statistical feature kurtosis (F.stat.kurt), with an importance value of 0.11. All other radiomics features and age showed importance values of < 0.08 (Figure S2a). In the radiomics‐only model, the statistical feature F.stat.kurt was the most impactful predictor (importance value, 0.33), followed by the textural features normalized gray level non‐uniformity from the size zone matrix (F_szm.glu.norm) and second measure of information theoretic correlation (F_cm.info.corr.2) (importance values of 0.083 and 0.076, respectively) (Appendix S6, Figures S2b and S3).

The performance of the radiomics‐only model, clinical–radiomics model, ADNEX model and subjective assessment in the training and validation sets is reported in Table 1 and Figure S4. In the training set, at a 10% risk‐of‐malignancy cut‐off, the AUC was 0.90 (95% CI, 0.89–0.92) for the radiomics‐only model, 0.96 (95% CI, 0.95–0.97) for the clinical–radiomics model and 0.93 (95% CI, 0.92–0.94) for the ADNEX model.

**Table 1:** Diagnostic performance of the Assessment of Different NEoplasias in the adneXa (ADNEX) model, radiomics‐only model, clinical–radiomics model and subjective assessment in training (*n* = 1451) and validation (*n* = 622) sets to classify malignant adnexal masses

	Model					
Parameter	ADNEX (10% cut‐off)	Radiomics‐only* (10% cut‐off)	Clinical–radiomics* (10% cut‐off)	Radiomics‐only* (41% cut‐off†)	Clinical–radiomics* (31% cut‐off†)	Subjective assessment
Training set						
AUC	0.93 (0.92–0.94)	0.90 (0.89–0.92)	0.96 (0.95–0.97)	0.90 (0.89–0.92)	0.96 (0.95–0.97)	NA‡
Accuracy	0.77 (0.75–0.79)	0.58 (0.55–0.60)	0.68 (0.65–0.70)	0.83 (0.81–0.85)	0.88 (0.87–0.90)	0.87 (0.85–0.89)
Sensitivity	0.95 (0.93–0.97)	0.99 (0.98–1)	0.99 (0.98–1)	0.83 (0.80–0.86)	0.92 (0.90–0.94)	0.95 (0.93–0.97)
Specificity	0.66 (0.63–0.69)	0.33 (0.30–0.36)	0.49 (0.46–0.52)	0.82 (0.80–0.85)	0.86 (0.84–0.88)	0.82 (0.80–0.85)
PPV	0.63 (0.60–0.66)	0.47 (0.44–0.50)	0.54 (0.51–0.57)	0.74 (0.71–0.78)	0.80 (0.77–0.83)	0.76 (0.73–0.80)
NPV	0.96 (0.94–0.97)	0.98 (0.96–0.99)	0.99 (0.98–1)	0.89 (0.87–0.91)	0.95 (0.93–0.96)	0.96 (0.95–0.98)
LR+	2.81 (2.55–3.07)	1.48 (1.41–1.55)	1.94 (1.81–2.06)	4.70 (4.01–5.39)	6.50 (5.44–7.55)	5.29 (4.55–6.04)
LR−	0.07 (0.04–0.10)	0.04 (0.01–0.07)	0.02 (0.01–0.05)	0.21 (0.17–0.25)	0.09 (0.06–0.12)	0.06 (0.04–0.08)
Validation set						
AUC	0.95 (0.93–0.97)	0.81 (0.78–0.84)	0.89 (0.86–0.92)	0.81 (0.78–0.84)	0.89 (0.86–0.92)	NA‡
Accuracy	0.82 (0.79–0.86)	0.58 (0.54–0.62)	0.67 (0.63–0.70)	0.75 (0.72–0.79)	0.82 (0.79–0.85)	0.90 (0.88–0.92)
Sensitivity	0.97 (0.95–0.99)	0.96 (0.94–0.98)	0.94 (0.91–0.97)	0.73 (0.68–0.79)	0.83 (0.79–0.88)	0.96 (0.94–0.98)
Specificity	0.72 (0.68–0.77)	0.32 (0.27–0.37)	0.48 (0.42–0.53)	0.77 (0.72–0.81)	0.81 (0.77–0.85)	0.86 (0.82–0.89)
PPV	0.71 (0.66–0.75)	0.49 (0.45–0.54)	0.55 (0.51–0.60)	0.68 (0.63–0.74)	0.75 (0.70–0.80)	0.82 (0.78–0.87)
NPV	0.97 (0.95–0.99)	0.92 (0.87–0.97)	0.92 (0.88–0.96)	0.81 (0.77–0.85)	0.88 (0.84–0.91)	0.97 (0.95–0.99)
LR+	3.49 (2.91–4.08)	1.41 (1.31–1.51)	1.79 (1.61–1.98)	3.13 (2.51–3.76)	4.39 (3.43–5.34)	6.80 (5.08–8.52)
LR−	0.04 (0.01–0.07)	0.12 (0.04–0.20)	0.12 (0.06–0.19)	0.35 (0.28–0.42)	0.20 (0.15–0.26)	0.05 (0.02–0.07)

Values in parentheses are 95% CI.

* Model trained using eXtreme Gradient Boosting (XGBoost).

† Cut‐off based on Youden's index.

‡ Area under receiver‐operating‐characteristics curve (AUC) for subjective assessment is not applicable (NA) as it is a binary judgment, not a probabilistic model. LR+, positive likelihood ratio; LR−, negative likelihood ratio; NPV, negative predictive value; PPV, positive predictive value.

The discriminative performance of both radiomics models deteriorated in the validation set, in which the radiomics‐only model showed the greatest deterioration in AUC. The radiomics features that contributed most to this effect were the textural features normalized gray level non‐uniformity from the run length matrix (F_rlm.glnu.norm) and F_cm.info.corr.2. High values of both these features represent heterogeneous echogenicity (Table S9). In the validation set, the AUC was 0.81 (95% CI, 0.78–0.84) for the radiomics‐only model, 0.89 (95% CI, 0.86–0.92) for the clinical–radiomics model and 0.95 (95% CI, 0.93–0.97) for the ADNEX model. The AUC of the ADNEX model was significantly higher than that of the radiomics‐only model (*P* < 0.001) and the clinical–radiomics model (*P* < 0.001). In the validation set, applying the 10% risk‐of‐malignancy cut‐off recommended for clinical use of the ADNEX model, the ADNEX model had a sensitivity of 0.97 (95% CI, 0.95–0.99) and a specificity of 0.72 (95% CI, 0.68–0.77), while the corresponding values for the clinical–radiomics model were 0.94 (95% CI, 0.91–0.97) and 0.48 (95% CI, 0.42–0.53), and for the radiomics‐only model they were 0.96 (95% CI, 0.94–0.98) and 0.32 (95% CI, 0.27–0.37). For the clinical–radiomics model, sensitivity and specificity were 0.83 (95% CI, 0.79–0.88) and 0.81 (95% CI, 0.77–0.85), respectively, at the optimal cut‐off of 31% based on Youden's index. For the radiomics‐only model, the corresponding values were 0.73 (95% CI, 0.68–0.79) and 0.77 (95% CI, 0.72–0.81) at the optimal cut‐off of 41% based on Youden's index. The sensitivity and specificity of subjective assessment by the original ultrasound examiner were 0.96 (95% CI, 0.94–0.98) and 0.86 (95% CI, 0.82–0.89) in the validation set.

Additional feature‐selection strategies (MRMR and RFE) were applied to the radiomics‐only XGBoost model to evaluate whether comparable performance could be obtained using alternative feature‐selection approaches (Table S10). Although the MRMR‐based model exhibited a slightly lower degree of overfitting, the feature‐selection approach combining the Mann–Whitney *U*‐test with Benjamini–Hochberg correction achieved the highest overall validation performance and was therefore chosen as the final method.

**Figure 2:**
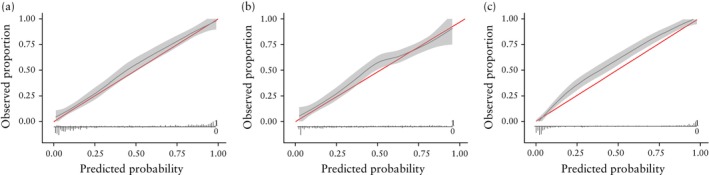
Calibration curves for prediction of malignant adnexal mass by clinical–radiomics (a), radiomics‐only (b) and Assessment of Different NEoplasias in the adneXa (ADNEX) (c) models in the validation set (*n* = 622, including 254 malignancies). Gray shading represents 95% CI. Red line indicates perfect calibration. Histograms within graphs illustrate distribution of predicted probability intervals, showing number of patients at each predicted probability who had a benign (0) or malignant (1) mass.

Calibration curves for each model are shown in Figure 2. The clinical–radiomics model was not optimally calibrated but appeared to be better calibrated than the radiomics‐only model and the ADNEX model in the validation set, with an intercept of 0.20 (95% CI, −0.02 to 0.43) and a slope of 0.93 (95% CI, 0.79–1.07). The calibration curves show that the ADNEX model consistently underestimated the risk of malignancy, except for the very lowest (< 0.08) and very highest (> 0.92) calculated risks. The clinical–radiomics model is better calibrated than the ADNEX model over the full range of calculated risks, and the radiomics‐only model is also better calibrated than the ADNEX model, except for at intermediate risks (38–54%).

**Figure 3:**
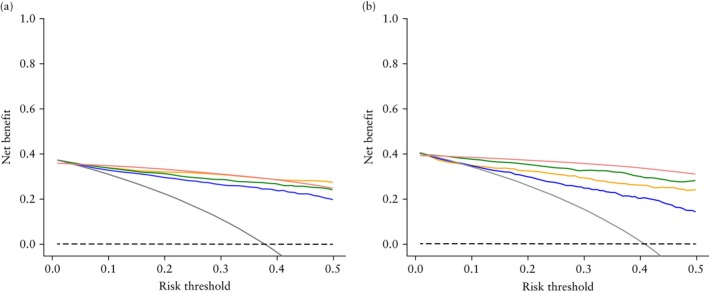
Decision curves for training (*n =* 1451, including 549 malignancies) (a) and validation (*n* = 622, including 254 malignancies) (b) sets, showing net benefit (number of true positives identified per patient after accounting for the harm of false positives at a given threshold) of using a specific model across a range of threshold probabilities (level of risk at which a clinician would choose to treat (refer patient to an oncology center)). Model is considered clinically useful when its net benefit curve lies above the treat‐all (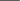) and treat‐none (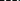) strategies across clinically relevant threshold probabilities. 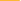, clinical–radiomics model; 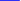, radiomics model; 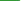, Assessment of Different NEoplasias in the adneXa (ADNEX) model; 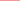, subjective assessment.

Decision curves for the training and validation sets are shown in Figure 3. In the validation set, the ADNEX model and subjective assessment showed clinical utility compared with alternative strategies at risk thresholds of approximately ≥ 3%, and the radiomics models showed clinical utility at risk thresholds of approximately ≥ 8%. For all risk thresholds from 5% to 50%, subjective assessment showed the highest net benefit, followed by the ADNEX model.

## DISCUSSION

We have shown that radiomics features differ between benign and malignant adnexal masses and have developed machine‐learning models that incorporate radiomics features to differentiate between benign and malignant adnexal masses in a large cohort of patients. In the developed models, among the radiomics features, F.stat.kurt emerged as the most influential predictor, possibly reflecting greater heterogeneity in malignant‐tissue structure.

The clinical–radiomics model that included age, serum CA 125 level and radiomics features outperformed the model based solely on radiomics features. The discriminative performance of both radiomics models deteriorated in the validation set, suggesting that both models may have been overfitted, and their discriminative performance was inferior to that of the ADNEX model. The clinical–radiomics model was better calibrated than the ADNEX model but exhibited lower clinical utility.

To the best of our knowledge, this is the largest study on radiomics analysis aimed at estimating the risk of malignancy of adnexal masses using ultrasound images^38^. Key strengths of the study include the use of a large, consecutively recruited patient cohort and the diversity of benign and malignant lesions in terms of histopathological types (Table S6). Important limitations include the single‐center study design, that the study was performed in a referral center for gynecological oncology, the exclusion of a large proportion of cases and the absence of external validation. This limits the generalizability of our findings. Additionally, exclusions may not have been random, introducing a risk of selection bias. Patients were excluded owing to unavailable images or images unsuitable for radiomics analysis (e.g. images with calipers or text on the lesion, or only partial tumor capture). It could be hypothesized that images with calipers and text were more likely to be saved than images without, and that more complex masses or masses suspicious for malignancy were more likely to be marked with calipers and text than less complex and benign‐looking masses. Indeed, malignancy was suspected more often by the original ultrasound examiner and there were more histologically confirmed malignant masses among the excluded patients than among the included patients. However, with the exception of more tumors with acoustic shadowing in the included group, there were only small differences in ultrasound variables between included and excluded tumors. In addition, a temporal imbalance was observed between included and excluded patients, with a larger proportion of included cases collected in more recent years. This may have introduced additional bias related to image quality, as more recent images would have been acquired using modern ultrasound machines equipped with advanced software, and related to the patient population, owing to variation in referral criteria, demographic characteristics or disease prevalence.

Another limitation of our study is that only one ultrasound image per tumor was used for radiomics analysis, primarily owing to the retrospective nature of the study. In most cases (74%), only one image per tumor met the criteria for radiomics analysis. The selected image may not have fully captured tumor heterogeneity. This is particularly relevant when comparing our radiomics models with the ADNEX model. During clinical application of the ADNEX model, the examiner evaluates multiple ultrasound images in real time. The radiomics model might have performed similarly or better than the ADNEX model did if multiple images per tumor had been analyzed. Although a subset of patients (540/2073 (26%)) had two or more available images suitable for radiomics analysis, we did not perform a subanalysis in this subgroup to assess whether incorporating multiple images could enhance model performance. This variable should be addressed in future research.

The manual segmentation of the ultrasound images by five different examiners may have introduced variability into the results of the radiomics analysis. However, we believe the effect of this to be small, since all five examiners received standardized training before performing segmentation and had access to a reference image indicating the borders of the adnexal mass, and all segmentations were revised and either approved or resegmented by a single EFSUMB Level‐II examiner before being used for radiomics analysis. Another limitation of the study is that we did not use three‐dimensional ultrasound volumes, meaning that morphological radiomics features, such as area, elongation and roughness, could not be evaluated. Additionally, in the validation set, the radiomics‐only model showed greater deterioration in discriminative performance than did the clinical–radiomics model. This may be related to the specific feature‐selection method that we employed. In machine learning it is useful to explore different feature‐selection strategies to mitigate overfitting while maintaining model performance, and this was addressed in the present study by evaluating multiple approaches.

Other research groups have developed ultrasound‐based radiomics machine‐learning models to differentiate between benign and malignant adnexal masses. The retrospective study of Liu *et al*.^19^ examined 1080 ovarian masses in 1080 patients and developed both a radiomics‐only model and a clinical–radiomics model. The latter included information on age, maximum diameter of the lesion, presence of symptoms, menopausal status and presence of ascites. The AUC of their radiomics‐only model was superior to that of ours (0.87 *vs* 0.81) while the AUC of their clinical–radiomics model was similar to that of ours (0.90 *vs* 0.89). Their clinical–radiomics model had superior discriminatory performance to that of their radiomics‐only model (AUC, 0.90 *vs* 0.87), however the statistical significance of this difference was not reported. Similarly, Barcroft *et al*.^13^ analyzed 1444 ultrasound images obtained from 761 patients, and developed and externally end‐to‐end validated a radiomics‐based model capable of automatically segmenting adnexal masses and estimating their risk of malignancy. In the end‐to‐end system, the entire process, from image acquisition to making predictions, was performed in a seamless, automated flow, with no manual intervention required at any stage. The discriminative performance of their model (AUC, 0.90) was comparable to the previously reported performance of expert subjective assessment (AUC, 0.96), for which the latter was calculated according to six levels of diagnostic confidence: certainly benign; probably benign; uncertain, but most likely benign; uncertain, but most likely malignant; probably malignant; and certainly malignant^39^. In a previous study by our group, we analyzed 775 preoperative ultrasound images obtained from 326 patients with an adnexal mass with solid ultrasound morphology (training set, *n* = 228; validation set, *n* = 98)^12^. It was found that the radiomics‐only model demonstrated poorer diagnostic performance in the validation set compared with the ADNEX model (AUC, 0.80 *vs* 0.88) and subjective assessment; the radiomics‐only model had a sensitivity of 0.78 and a specificity of 0.76 at the optimal 68% risk‐of‐malignancy cut‐off, while subjective assessment had a sensitivity of 0.99 and a specificity of 0.72.

An international consensus statement^6^ recommends a 10% risk threshold to refer patients with an adnexal mass to an oncology center, accepting that nine patients with a benign mass may be referred unnecessarily per each correctly referred patient with a malignant mass. However, at a 10% risk‐of‐malignancy cut‐off, our radiomics models offered little advantage over referring all patients (Figure 3)^40^. In our clinical–radiomics model, the ultrasound‐based information included in the ADNEX model is replaced by radiomics‐derived features. It is evident that, in the current study, radiomics data were not a suitable replacement for the ultrasound data included in the ADNEX model, and the radiomics models performed worse than both the ADNEX model and subjective assessment. The inferior performance of our clinical–radiomics models compared with the ADNEX model is probably explained by radiomics containing ultrasound information that is completely different from that included in the ADNEX model. The ADNEX model considers morphological features, such as papillary projections and number of cyst locules, whereas our radiomics models analyze pixels. Moreover, the variables in the ADNEX model were obtained during live scanning, meaning that information from the whole tumor was available, rather than information from only a single image. The original ultrasound examiner, performing subjective assessment, also had access to color‐ and power‐Doppler ultrasound data. This suggests that a radiomics model could potentially be improved by using more than one image or using ultrasound volumes for analysis, the latter providing the possibility of assessing more features (e.g. volume, surface area, sphericity, compactness, elongation, roughness, curvature measures) and better capture tumor heterogeneity.

It would be of significant clinical value to develop a machine‐learning model that can discriminate between benign and malignant adnexal masses with a performance similar to that of subjective assessment by an experienced examiner or the ADNEX model. Unlike both subjective assessment and the ADNEX model, radiomics models are quantitative tools that require only ultrasound images to estimate the risk of malignancy, with no need for expert interpretation. However, they rely on the ability of the ultrasound examiner to produce representative ultrasound images, whereas the ADNEX model only requires the examiner to have some level of ultrasound expertise and familiarity with IOTA terminology. Radiomics software is currently not integrated into commercially available ultrasound machines; it is used only for scientific purposes and requires manual export and conversion of images before analysis. Manual segmentation of the ROI typically takes a few minutes and once the ROI is delineated, feature extraction and model inference are fully automated and completed within seconds. For clinical use, automated segmentation is needed to enable end‐to‐end automation. If improvements in the discriminative performance of radiomics models to differentiate between benign and malignant adnexal masses can be achieved, and if automated‐segmentation radiomics models can be successfully integrated into ultrasound systems, radiomics analysis could support less experienced examiners by providing immediate results based on representative ultrasound images. However, the integration of radiomics into clinical ultrasound systems faces practical challenges, such as computational demands for image processing and feature extraction, the need for workflow standardization (e.g. deciding which type of image to acquire: DICOM or JPEG; two‐dimensional images or ultrasound volumes; how many images per tumor to analyze) and ensuring compatibility across different types of ultrasound machine. Moreover, approval of radiomics software as a medical device would be required^41^. In addition to improving the discriminative performance of radiomics models for benign *vs* malignant adnexal masses, future studies could explore the effect of integrating radiomics features into the ADNEX model.

In conclusion, our radiomics models cannot be recommended for clinical application. However, our results support that radiomics analysis of ultrasound images of adnexal masses has potential and warrants further investigation. Future studies should include a larger number of ultrasound images per tumor (at least two) or ultrasound volumes to capture the spatial heterogeneity of a tumor, which may improve the performance of radiomics models. To justify the clinical implementation of radiomics models, the aim would be to achieve an AUC similar to that of the ADNEX model (AUC, 0.93–0.95).

## Supporting information


**Appendix S1** Information regarding International Ovarian Tumor Analysis (IOTA) database and IOTA phase‐5 and IOTA phase‐7 studies.
**Appendix S2** Details of ultrasound machines used.
**Appendix S3** Statistical and textural radiomics features.
**Appendix S4** Description of International Ovarian Tumor Analysis **(**IOTA) Assessment of Different NEoplasias in the adneXa (ADNEX) model.
**Appendix S5** Radiomics features (*n* = 14) that differed significantly between benign and malignant tumors in the training set (*n* = 1451) and were included in both radiomics models.
**Appendix S6** Most important radiomics features, identified by feature importance analysis.
**Table S1** Distribution of ultrasound machines used in training and validation sets.
**Table S2** Clinical and ultrasound characteristics of patients included and excluded in final analysis.
**Table S3** Years in which ultrasound examinations were performed, for all eligible patients and according to inclusion status.
**Table S4** Number of available ultrasound images per patient.
**Table S5** Clinical and ultrasound characteristics of patients in training set (*n* = 1451).
**Table S6** Clinical and ultrasound characteristics of patients in validation set (*n* = 622).
**Table S7** Histological outcomes of patients in training and validations sets.
**Table S8** Distribution of tumor histology and International Federation of Gynaecology and Obstetric (FIGO) stages (*n* = 2073).
**Table S9** SHapley Additive exPlanations (SHAP) analysis of feature importance in the radiomics‐only model.
**Table S10** Comparison of performance of radiomics‐only model using different feature selection methods.
**Figure S1** Principal Component Analysis (PCA) of radiomics features in training set.
**Figure S2** Ranking of feature importance in (a) clinical–radiomics model and (b) radiomics‐only model.
**Figure S3** Distribution of kurtosis (F.stat.kurt) feature in benign and malignant masses.
**Figure S4** Receiver‐operating‐characteristics (ROC) curves for radiomics‐only, clinical–radiomics and Assessment of Different NEoplasias in the adneXa (ADNEX) models in (a) training set (*n* = 1451, of which 549 (37.83%) were malignant) and (b) validation set (*n* = 622, of which 254 (40.84%) were malignant).

## Data Availability

The data that support the findings of this study are available from the corresponding author upon reasonable request.
