# Supplementary material for: Developing and validating ultrasound‐based machine‐learning models incorporating radiomics features to predict malignancy in adnexal masses
Source: Ultrasound Obstet Gynecol. 2026 Mar 30;67(4):519–29. doi: 10.1002/uog.70203 (PMC13040134; doi:10.1002/uog.70203)
Supplement: Supplementary file 1 — Appendix S1 Information regarding International Ovarian Tumor Analysis (IOTA) database and IOTA phase‐5 and IOTA phase‐7 studies. Appendix S2 Details of ultrasound machines used. Appendix S3 Statistical and textural radiomics features. Appendix S4 Description of International Ovarian Tumor Analysis (IOTA) Assessment of Different NEoplasias in the adneXa (ADNEX) model. Appendix S5 Radiomics features (n = 14) that differed significantly between benign and malignant tumors in the training set (n = 1451) and were included in both radiomics models. Appendix S6 Most important radiomics features, identified by feature importance analysis. Table S1 Distribution of ultrasound machines used in training and validation sets. Table S2 Clinical and ultrasound characteristics of patients included and excluded in final analysis. Table S3 Years in which ultrasound examinations were performed, for all eligible patients and according to inclusion status. Table S4 Number of available ultrasound images per patient. Table S5 Clinical and ultrasound characteristics of patients in training set (n = 1451). Table S6 Clinical and ultrasound characteristics of patients in validation set (n = 622). Table S7 Histological outcomes of patients in training and validations sets. Table S8 Distribution of tumor histology and International Federation of Gynaecology and Obstetric (FIGO) stages (n = 2073). Table S9 SHapley Additive exPlanations (SHAP) analysis of feature importance in the radiomics‐only model. Table S10 Comparison of performance of radiomics‐only model using different feature selection methods. Figure S1 Principal Component Analysis (PCA) of radiomics features in training set. Figure S2 Ranking of feature importance in (a) clinical–radiomics model and (b) radiomics‐only model. Figure S3 Distribution of kurtosis (F.stat.kurt) feature in benign and malignant masses. Figure S4 Receiver‐operating‐characteristics (ROC) curves for radiomics‐only, clinical–radiomics and Assessment of Different [file UOG-67-519-s001.docx]

**Developing and validating ultrasound-based machine learning models incorporating radiomics features to predict malignancy in adnexal masses**

**Supplementary material**

**Appendix S1: IOTA database, IOTA phase 5 and 7**

The IOTA database is a research database that includes all patients in the international multicenter IOTA studies. It contains clinical, ultrasound and histological data of patients with an adnexal mass examined with transvaginal (or transrectal) ultrasound, supplemented with abdominal ultrasound if needed, before surgery and follow-up data for non-surgically managed patients. The IOTA phase 7 study (unpublished) was designed to create and validate risk prediction models to discriminate between benign and malignant adnexal tumors and to recruit consecutive patients with at least one adnexal mass on ultrasound that was removed by surgery, while the primary aim of the IOTA 5 study was to elucidate the natural history of adnexal masses with benign ultrasound morphology managed conservatively with regular follow up ^1^.

**Appendix S2: Ultrasound machines used**

Voluson E10 (GE Healthcare, Zipf, Austria); Samsung HERA W10, Samsung HERA HS70A, Samsung HERA WS80 and Samsung HERA I10 (Samsung Medison Co. Ltd., Seoul, South Korea); Canon Aplio i700 and Canon Aplio i900 (Canon Medical Systems Europe BV, Zoetermeer, The Netherlands).

**Appendix S3: Families of radiomics features**

The statistical features describe the statistical properties of the grey level histogram within the Region Of Interest (ROI) (e.g. mean, median, variance, skewness, kurtosis), while the textural features provide information on the spatial distribution of the grey levels within the ROI.

The textural features are computed based on the following matrixes: 1) grey level size zone matrix (F_szm), which represents the number of groups (zones) of neighboring pixels with the same gray level; 2) grey level run length matrix (F_rlm), which represents the length of a consecutive sequence of pixels with the same grey level; 3) grey level co-occurrence matrix (F_cm), in which the co- occurrence of gray levels with neighboring pixels is reported ^2^.

**Appendix S4: IOTA ADNEX model**

IOTA ADNEX model is based on three clinical and six ultrasound features: age of the patient (in years), serum CA125 (U/mL), maximum diameter of the lesion (in mm; *‘mdl’*), the proportion of solid tissue calculated as the maximum diameter of the largest solid component in mm divided by the maximum diameter of the lesion in mm (value between 0 and 1; *‘pst’*), presence of more than 10 cyst locules (1 versus 0; *‘tcl’*), the number of papillary projections (0, 1, 2, 3, 4, with 4 indicating more than three; *‘nps’*), presence of acoustic shadows (1 versus 0; *‘sha’*), the presence of ascites (1 versus 0; *‘asc’*), and examination at an oncology center (1 versus 0; *‘oc’*).

The ADNEX model can be calculated both with and without information on CA 125, using the following formulas ^3^:

Risk benign= 1/(1+ exp(z1)+ exp(z2)+ exp(z3)+ exp(z4))

Risk borderline= exp(z1)/(1+ exp(z1)+ exp(z2)+ exp(z3)+ exp(z4))

Risk stage 1 cancer= exp(z2)/(1+ exp(z1)+ exp(z2)+ exp(z3)+ exp(z4))

Risk stage 2-4 cancer = exp(z3)/(1+ exp(z1)+ exp(z2)+ exp(z3)+ exp(z4)

Risk secondary metastasis= exp(z4)/(1+ exp(z1)+ exp(z2)+ exp(z3)+ exp(z4))

Where, for ADNEX **with CA125:**

z1= -7,577663+0,004506 * age + 0,111642 * log2(CA125) + 0,372046 * log2( mdl) + 6,967853 * pst - 5,65588 * pst2 + 1,375079 * tcl + 0,604238 * nps - 2,04157 * sha + 0,971061 * asc + 0,953043 * onc

z2= -12,276041 + 0,01726 * age + 0,197249 * log2(CA125) + 0,87353 * log2( mdl) + 9,583053 * pst - 5,83319 *pst2 + 0,791873 * tcl + 0,400369 * nps - 1,87763 * sha + 0,452731 * asc + 0,452484 * onc

z3= -14,91583 + 0,051239 * age + 0,765456 * log2(CA125) +0,430477 * log2( mdl) + 10,37696 * pst - 5,70975 *pst2 + 0,273692 * tcl + 0,389874 * nps - 2,35516 * sha + 1,348408 * asc + 0,459021 * onc

z4= -11,909267 + 0,033601 * age + 0,276166 * log2(CA125) +0,449025 * log2( mdl) + 6,644939 * pst - 2,3033 *pst2 + 0,89998 * tcl +0,215645 * nps - 2,49845 * sha + 1,636407 * asc + 0,808887 * onc

While, for ADNEX **without CA125:**

z1= -7,412534 + 0,003489 * age + 0,430701 * log2( mdl) + 7,117925 * pst - 5,74135 *pst2 + 1,343699 * tcl + 0,607211 * nps - 2,11885 * sha + 1,167767 * asc + 0,983227 * onc

z2= -12,201607 + 0,017607 * age + 0,98728 * log2( mdl) + 10,07145 * pst - 6,17742 *pst2 + 0,763081 * tcl + 0,410449 * nps - 1,98073 * sha + 0,77054 * asc + 0,543677 * onc

z3= -12,826207 + 0,045172 * age + 0,759002 * log2( mdl) + 11,83296 * pst - 6,64336 *pst2 + 0,316444 * tcl + 0,390959 * nps - 2,94082 * sha + 2,691276 * asc + 0,929483 * onc

z4= -11,424379 + 0,033407 * age + 0,560396 * log2( mdl) + 7,264105 * pst - 2,77392 *pst2 + 0,983394 * tcl +0,199164 * nps - 2,63702 * sha + 2,185574 * asc + 0,906249 * onc

**Appendix S5: Radiomics features (*n*= 14) that differed significantly between benign and malignant tumors in the training set (n = 1,451) and were included in both radiomics models**

(‘F_stat.kurt’, ‘F_stat.entropy’, ‘F_cm.inv.diff.mom.norm’, ‘F_cm.auto.corr’, ‘F_cm.clust.tend’, ‘F_cm.info.corr.2’, ‘F_cm.inv.var’, F_rlm.glnu’, F_rlm.glnu.norm’, ‘F_rlm.rl.var’, ‘F_szm.lzhge’, ‘F_szm.glnu’, ‘F_szm.glnu.norm’, F_szm.zsnu.norm’)

**Appendix S6: Most important radiomics features identified by the feature importance analysis**

The feature ‘F.stat.kurt’ (kurtosis) describes how pixel intensities are spread around the mean gray-level value within a ROI (in this case, ovarian lesion), i.e. whether pixel values are mostly similar or vary widely. A high kurtosis value means that the distribution has heavier tails and a more pronounced peak, indicating that the lesion appears relatively uniform, whereas a low kurtosis value indicates that the pixel intensities are evenly distributed around the mean, with a flatter distribution, corresponding to a more heterogeneous appearance of the mass (Figure S3). ‘F_szm.glnu.norm’ (normalized grey level non-uniformity from the size zone matrix (SZM)) measures the variation of gray-level values across connected areas within the lesion. A high value indicates that the lesion has uneven pixel intensities, reflecting a heterogeneous tumor texture on ultrasound, whereas a low value indicates greater similarity in intensity values, corresponding to a homogeneous tumor texture on ultrasound. ‘F_cm.info.corr.2’ (second measure of information theoretic correlation) is a textural feature belonging to the class of gray-level co-occurrence matrix (GLCM) features based on information theory. It relates to the randomness of data, quantifying the complexity of the texture of the tumor. Low values (low information) correspond to a uniform or plain texture, while high values (high information) indicate that tumor texture is more complex and varied.

**Table S1.** Distribution of ultrasound machines used in training and validation sets.

| **Scanner** | **Train set**  **(n= 1,451)** | **Validation set**  **(n=622)** |
| --- | --- | --- |
| **Samsung** (n, %) | 980 (67.53 %) | 390 (62.70 %) |
| **GE** (n, %) | 450 (31 %) | 222 (35.69 %) |
| **Philips** (n, %) | 8 (0.55 %) | 6 (0.96 %) |
| **Toshiba** (n, %) | 7 (0.48 %) | 1 (0.16 %) |
| **Canon** (n, %) | 5 (0.34 %) | 2 (0.32 %) |
| **Siemens** (n, %) | 1 (0.07 %) | 0 |
| **Esaote** (n, %) | 0 | 1 (0.16 %) |

**Table S2.** Clinical and ultrasound characteristics of patients included in the final analysis and those of excluded patients.

|  | **Total study population**  **(n= 2,073)** | **Excluded**  **(n = 2,428)** | **P value** |
| --- | --- | --- | --- |
| *Clinical variables* |  | | |
| **AGE** (median, IQR) | 51 (39-61) | 51 (39-62) | 0.48 |
| **MENOPAUSAL STATUS** (n, %)  Pre  Post | 1010 (48.7)  1063 (51.3) | 1211 (49.9)  1217 (50.1) | 0.44 |
| **PARITY** (n, %)  0  ≥1 | 840 (40.5)  1233 (59.5) | 971 (40.0)  1457 (60.0) | 0.72 |
| **SYMPTOMS** (n, %)  Yes  No | 863 (41.6)  1210 (58.4) | 1104 (45.5)  1324 (54.5) | 0.010 |
| **HISTOLOGY** (n, %)  Benign  Borderline  Early stage  Advanced stage  Metastatic | 1270 (61.3)  113 (5.4)  268 (12.9)  325 (15.7)  97 (4.7) | 1251 (51.5)  231 (9.6)  226 (9.3)  569 (23.4)  151 (6.2) | <0.0001 |
| **FIGO for primary invasive early stage and advanced tumors** (n, %)  FIGO I  FIGO II  FIGO III  FIGO IV | 202 (34.1)  66 (11.1)  230 (38.8)  95 (16.0) | 172 (21.6)  54 (6.8)  431 (54.2)  138 (17.4) | <0.0001 |
| *Ultrasound variables* |  | | |
| **LATERALITY** (n, %)  Unilateral  Bilateral | 1,497 (72.2)  576 (27.8) | 1,621 (66.8)  807 (33.2) | <0.001 |
| **SIDE** (n, %)  Central  Left  Right | 187 (9.0)  924 (44.6)  962 (46.4) | 292 (12.0)  1,047 (43.1)  1,089 (44.9) | 0.005 |
| **ORIGIN** (n, %)  Fallopian tube  Ovary  Paraovarian  Uncertain | 49 (2.4)  1,880 (90.7)  40 (1.9)  104 (5.0) | 63 (2.6)  2,210 (91.0)  25 (1.0)  130 (5.4) | 0.080 |
| **SUBJECTIVE ASSESSMENT** (n, %)  Benign  Borderline  Malignant | 1,093 (52.7)  215 (10.4)  765 (36.9) | 1,047 (43.1)  304 (12.5)  1,077 (44.4) | <0.001 |
| **CERTAINTY of subjective assessment** (n, %)  Certainly benign  Probably benign  Uncertain  Probably malignant  Certainly malignant | 595 (28.7)  464 (22.4)  110 (5.3)  289 (13.9)  615 (29.7) | 606 (25.0)  395 (16.3)  116 (4.8)  421 (17.3)  890 (36.7) | <0.001 |
| **Max diameter of lesion** (median, IQR) | 65 (44-100) | 70 (45-110) | 0.001 |
| **Max diameter of solid component**  (median, IQR) | 17 (0-55) | 24 (0-58) | 0.008 |
| **More than 10 cyst locules** (n, %)  No  Yes | 1,792 (86.4)  281 (13.6) | 2,074 (85.4)  354 (14.6) | 0.32 |
| **Number of papillary projections** (n, %)  0  1  2  3  4 | 1,613 (77.8)  216 (10.4)  57 (2.7)  41 (2.0)  146 (7.0) | 1,970 (81.1)  218 (9.0)  53 (2.2)  35 (1.4)  152 (6.3) | 0.08 |
| **Acoustic shadows** (n, %)  No  Yes | 1,613 (77.8)  460 (22.2) | 2,163 (89.1)  265 (10.9) | <0.001 |
| **Presence of ascites** (n, %)  No  Yes | 1,868 (90.1)  205 (9.9) | 2,029 (83.6)  399 (16.4) | <0.001 |
| **COLOR SCORE** (n, %)  1  2  3  4 | 792 (38.2)  517 (24.9)  469 (22.6)  295 (14.2) | 786 (32.4)  595 (24.5)  680 (28.0)  367 (15.1) | <0.001 |
| **CYST CONTENT** (n, %)  Anechoic  Ground glass  Hemorrhagic  Low level  Mixed  Not applicable | 469 (22.6)  213 (10.3)  9 (0.3)  561 (27.1)  236 (11.4)  585 (28.2) | 532 (21.9)  215 (8.9)  13 (0.5)  696 (28.7)  194 (8.0)  778 (32.0) | <0.001 |
| **KNOWN CA125 at inclusion** (n, %)  No  Yes | 521 (25.1)  1,552 (74.9) | 766 (31.5)  1,662 (68.5) | <0.001 |
| **CA125**  (median IQR) | 24 (10-119) | 39 (13-250) | <0.001 |

**Table S3.** Years in which ultrasound examinations were performed, for all eligible patients and according to inclusion status.

| **Year** | **Elegible patients**  **(n= 4,501) (n, %)** | **Included patients**  **(n= 2,073) (n, %)** | **Excluded patients**  **(n= 2,428) (n, %)** |
| --- | --- | --- | --- |
| **2012** | 98 (2.18) | 1 (0.05) | 97 (4.00) |
| **2013** | 186 (4.13) | 2 (0.10) | 184 (7.58) |
| **2014** | 122 (2.71) | 19 (0.92) | 103 (4.24) |
| **2015** | 175 (389) | 25 (1.21) | 150 (6.18) |
| **2016** | 82 (1.82) | 31 (1.50) | 51 (2.10) |
| **2017** | 296 (6.58) | 82 (3.96) | 214 (8.81) |
| **2018** | 424 (9.42) | 64 (3.09) | 360 (14.83) |
| **2019** | 631 (14.02) | 258 (12.45) | 373 (15.36) |
| **2020** | 655 (14.55) | 298 (14.38) | 357 (14.70) |
| **2021** | 882 (19.60) | 529 (25.52) | 353 (14.54) |
| **2022** | 377 (8.38) | 269 (12.98) | 108 (4.45) |
| **2023** | 573 (12.73) | 495 (23.88) | 78 (3.21) |
| **Total** | 4,501 | 2,073 | 2,428 |

**Table S4.** Number of images per patient.

| **Type of image** | **Number of patients (n, %)** |
| --- | --- |
| **Gray scale without caliper or text (suitable for radiomics)**  1 image  2 images  3 images  >3 images | 1,533 (74.0)  300 (14.5)  115 (5.5)  125 (6.0) |
| **Total** | **2,073** |

**Table S5.** Clinical and ultrasound characteristics of patients in training set (*n* = 1451).

|  | **Total training set**  **(n= 1,451)** | **Benign**  **(n = 902)** | **Malignant (n= 549)** | | | | | **P value*** |
| --- | --- | --- | --- | --- | --- | --- | --- | --- |
|  |  |  | All malignant  (n= 549) | Borderline  (n=82) | Early stage  (FIGO I-II)  (n=176) | Advanced  (FIGO III-IV)  (n=225) | Metastatic  (n=66) |  |
| *Clinical variables* |  | | | | | | | |
| **AGE, years** (median, IQR) | 51 (39-61) | 48 (34-60) | 55 (46-64) | 42 (31-53) | 55 (47-63) | 58 (51-67) | 55 (45-64) | <0.001/<0.001 |
| **MENOPAUSAL STATUS** (n, %)  Pre  Post | 707 (48.7)  744 (51.3) | 508 (56.3)  394 (43.7) | 199 (36.2)  350 (63.8) | 55 (67.1)  27 (32.9) | 60 (34.1)  116 (65.9) | 59 (26.2)  166 (73.8) | 25 (37.9)  41 (62.1) | <0.001/<0.001 |
| **PARITY** (n, %)  0  ≥1 | 586 (40.4)  865 (59.6) | 408 (45.2)  494 (54.8) | 178 (32.4)  371 (67.6) | 46 (56.1)  36 (43.9) | 63 (35.8)  113 (64.2) | 55 (24.4)  170 (75.6) | 14 (21.2)  52 (78.8) | <0.001/<0.001 |
| **SYMPTOMS** (n, %)  Yes  No | 603 (41.6)  848 (58.4) | 274 (30.4)  628 (69.6) | 329 (59.9)  220 (40.1) | 36 (43.9)  46 (56.1) | 92 (52.3)  84 (47.7) | 159 (70.7)  66 (29.3) | 42 (63.6)  24 (36.4) | <0.001/0.080 |
| *Ultrasound variables* |  | | | | | | | |
| **LATERALITY** (n, %)  Unilateral  Bilateral | 1,033 (71.2)  418 (28.8) | 701 (77.7)  201 (22.3) | 332 (60.5)  217 (39.5) | 61 (74.4)  21 (25.6) | 136 (77.3)  40 (22.7) | 106 (47.1)  119 (52.9) | 29 (43.9)  37 (56.1) | <0.001/<0.001 |
| **SIDE** (n, %)  Central  Left  Right | 132 (9.1)  630 (43.4)  689 (47.5) | 36 (4.0)  420 (46.6)  446 (49.4) | 96 (17.5)  210 (38.3)  243 (44.3) | 5 (6.1)  35 (42.7)  42 (51.2) | 28 (15.9)  71 (40.3)  77 (43.8) | 55 (24.5)  79 (35.1)  91 (40.4) | 8 (12.1)  25 (37.9)  33 (50.0) | <0.001/0.009 |
| **ORIGIN** (n, %)  Fallopian tube  Ovary  Paraovarian  Uncertain | 26 (1.8)  1,336 (92.1)  23 (1.6)  66 (4.5) | 21 (2.3)  834 (92.5)  22 (2.4)  25 (2.8) | 5 (0.9)  502 (91.4)  1 (0.2)  41 (7.5) | 0  81 (98.8)  1 (1.2)  0 | 0  164 (93.2)  0  12 (6.8) | 4 (1.8)  199 (88.4)  0  22 (9.8) | 1 (1.5)  58 (87.9)  0  7 (10.6) | <0.001/0.020 |
| **SUBJECTIVE ASSESSMENT** (n, %)  Benign  Borderline  Malignant | 767 (52.9)  154 (10.6)  530 (36.5) | 740 (82.1)  87 (9.6)  75 (8.3) | 27 (4.9)  67 (12.2)  455 (82.9) | 10 (12.2)  42 (51.2)  30 (36.6) | 13 (7.4)  20 (11.4)  143 (81.3) | 3 (1.3)  3 (1.3)  219 (97.3) | 1 (1.5)  2 (3.0)  63 (95.5) | <0.001/<0.001 |
| **CERTAINTY of subjective assessment** (n, %)  Certainly benign  Probably benign  Uncertain  Probably malignant  Certainly malignant | 419 (28.9)  325 (22.4)  421 (29.0)  209 (14.4)  77 (5.3) | 403 (44.7)  312 (34.6)  60 (6.7)  91 (10.1)  36 (4.0) | 16 (2.9)  13 (2.4)  17 (3.1)  118 (21.5)  385 (70.1) | 3 (3.7)  8 (9.8)  6 (7.3)  44 (53.7)  21 (25.6) | 7 (4.0)  5 (2.8)  7 (4.0)  47 (26.7)  110 (62.5) | 4 (1.8)  0  1 (0.4)  23 (10.2)  197 (87.6) | 2 (3.0)  0  3 (4.5)  4 (6.1)  57 (86.4) | <0.001/<0.001 |
| **ADNEX - Max diameter of lesion**, **mm**(median, IQR) | 66  (44-101) | 56  (39-82) | 90  (59-134) | 75  (48-122) | 95  (57-143) | 89  (62-120) | 104  (73-160) | <0.001/0.013 |
| **ADNEX - Max diameter of solid component, mm**  (median, IQR) | 16  (0-54) | 0  (0-20) | 57  (31-84) | 22  (7-49) | 52  (29-81) | 66  (46-95) | 78  (48-100) | <0.001/<0.001 |
| **ADNEX - more than 10 cyst locules** (n, %)  No  Yes | 1,253 (86.4)  198 (13.6) | 823 (91.2)  79 (8.8) | 430 (78.3)  119 (21.7) | 65 (79.3)  17 (20.7) | 139 (79.0)  37 (21.0) | 182 (80.9)  43 (19.1) | 44 (66.7)  22 (33.3) | <0.001/0.10 |
| **ADNEX - Number of papillary projections** (n, %)  0  1  2  3  4 | 1,129 (77.8)  156 (10.7)  35 (2.4)  27 (1.9)  104 (7.2) | 733 (81.3)  117 (13.0)  23 (2.5)  12 (1.3)  17 (1.9) | 396 (72.1)  39 (7.1)  12 (2.2)  15 (2.7)  87 (15.8) | 38 (46.3)  14 (17.1)  3 (3.7)  2 (2.4)  25 (30.5) | 122 (69.3)  15 (8.5)  4 (2.3)  7 (4.0)  28 (15.9) | 178 (79.1)  10 (4.4)  4 (1.8)  5 (2.2)  28 (12.4) | 58 (87.9)  0  1 (1.5)  1 (1.5)  6 (9.1) | <0.001/<0.001 |
| **ADNEX - Acoustic shadows** (n, %)  No  Yes | 1,126 (77.6)  325 (22.4) | 622 (69.0)  280 (31.0) | 504 (91.8)  45 (8.2) | 74 (90.2)  8 (9.8) | 159 (90.3)  17 (9.7) | 210 (93.3)  15 (6.7) | 61 (92.4)  5 (7.6) | <0.001/0.68 |
| **ADNEX - Presence of ascites** (n, %)  No  Yes | 1310 (90.3)  141 (9.7) | 898 (99.6)  4 (0.4) | 412 (75.0)  137 (25.0) | 78 (95.1)  4 (4.9) | 160 (90.9)  16 (9.1) | 131 (58.2)  94 (41.8) | 43 (65.2)  23 (34.8) | <0.001/<0.001 |
| **COLOR SCORE** (n, %)  1  2  3  4 | 560 (38.6)  349 (24.1)  339 (23.4)  203 (14.0) | 530 (58.8)  249 (27.6)  105 (11.6)  18 (2.0) | 30 (5.5)  100 (18.2)  234 (42.6)  185 (33.7) | 13 (15.9)  35 (42.7)  25 (30.5)  9 (11.0) | 9 (5.1)  36 (20.5)  77 (43.8)  54 (30.7) | 7 (3.1)  24 (10.7)  101 (44.9)  93 (41.3) | 1 (1.5)  5 (7.6)  31 (47.0)  29 (43.9) | <0.001/<0.001 |
| **CYST CONTENT** (n, %)  Anechoic  Ground glass  Hemorrhagic  Low level  Mixed  Not applicable | 325 (22.4)  150 (10.3)  4 (0.3)  391 (26.9)  171 (11.8)  410 (28.3) | 258 (28.6)  113 (12.5)  1 (0.1)  219 (24.3)  160 (17.7)  151 (16.7) | 67 (12.2)  37 (6.7)  3 (0.5)  172 (31.3)  11 (2.0)  259 (47.2) | 9 (11.0)  7 (8.5)  0  45 (54.9)  3 (3.7)  18 (22.0) | 22 (12.5)  22 (12.5)  2 (1.1)  60 (34.1)  2 (1.1)  68 (38.6) | 32 (14.2)  7 (3.1)  1 (0.4)  45 (20.0)  4 (1.8)  136 (60.4) | 4 (6.1)  1 (1.5)  0  22 (33.3)  2 (3.0)  37 (56.1) | <0.001/<0.001 |
| **KNOWN CA125 at inclusion** (n, %)  No  Yes | 359 (24.7)  1092 (75.3) | 253 (28.0)  649 (72.0) | 106 (19.3)  443 (80.7) | 14 (17.1)  68 (82.9) | 32 (18.2)  144 (81.8) | 39 (17.3)  186 (82.7) | 21 (31.8)  45 (68.2) | <0.001/0.055 |
| **CA125**  (median IQR) | 25 (10-109) | 14  (9-30) | 126  (35-522) | 21  (11-61) | 62  (22-194) | 463  (149-1250) | 85  (42-360) | <0.001/<0.001 |

IQR, interquartile range; FIGO, International Federation of Gynecology and Obstetrics; ADNEX, Assessment of Different NEoplasias in the adnexa; CA, Cancer Antigen. * P-value was calculated with two-sided Pearson’s Chi-Square or Fisher’s Exact or Mann-Whitney or Kruskall-Wallis test as appropriate. The statistical level of significance was set at P<0.05. Of the two p-values reported, the first represents the staistical significance of the differences between benign and malignant tumors, whereas the second represents the statistical significance of the differences between the subtypes of ovarian malignancies (borderline, early stage, advanced and metastatic).

**Table S6.** Clinical and ultrasound characteristics of patients in validation set (*n*= 622).

|  | **Total validation set**  **(n=622)** | **Benign**  **(n= 368)** | **Malignant (n= 254)** | | | | | **P value*** |
| --- | --- | --- | --- | --- | --- | --- | --- | --- |
|  |  |  | All malignant  (n=254) | Borderline  (n=31) | Early stage  (FIGO I-II)  (n=92) | Advanced  (FIGO III-IV)  (n=100) | Metastatic  (n=31) |  |
| *Clinical variables* |  | | | | | | | |
| **AGE, years** (median, IQR) | 51 (39-60) | 48 (34-56) | 55 (47-65) | 47 (36-55) | 55 (46-67) | 57 (51-66) | 56 (51-64) | <0.001/0.001 |
| **MENOPAUSAL STATUS** (n, %)  Pre  Post | 303 (48.7)  319 (51.3) | 213 (57.9)  155 (42.1) | 90 (35.4)  164 (64.6) | 18 (58.1)  13 (41.9) | 35 (38.0)  57 (62.0) | 27 (27.0)  73 (73.0) | 10 (32.3)  21 (67.7) | <0.001/0.015 |
| **PARITY** (n, %)  0  ≥1 | 254 (40.8)  368 (59.2) | 171 (46.5)  197 (53.5) | 83 (32.7)  171 (67.3) | 14 (45.2)  17 (54.8) | 28 (30.4)  64 (69.6) | 37 (37.0)  63 (63.0) | 4 (12.9)  27 (87.1) | 0.001/0.033 |
| **SYMPTOMS** (n, %)  Yes  No | 260 (41.8)  362 (58.2) | 112 (30.4)  256 (69.6) | 148 (58.3)  106 (41.7) | 10 (32.3)  21 (67.7) | 53 (57.6)  39 (42.4) | 66 (66.0)  34 (34.0) | 19 (61.3)  12 (38.7) | <0.001/0.011 |
| *Ultrasound variables* |  | | | | | | | |
| **LATERALITY** (n, %)  Unilateral  Bilateral | 464 (74.6)  158 (25.4) | 305 (82.9)  63 (17.1) | 159 (62.6)  95 (37.4) | 25 (80.6)  6 (19.4) | 70 (76.1)  22 (23.9) | 45 (45.0)  55 (55.0) | 19 (61.3)  12 (38.7) | <0.001/0.001 |
| **SIDE** (n, %)  Central  Left  Right | 55 (8.8)  294 (47.3)  273 (43.9) | 14 (3.8)  193 (52.4)  161 (43.8) | 41 (16.2)  101 (39.8)  112 (44.1) | 3 (9.7)  14 (45.2)  14 (45.2) | 21 (22.8)  34 (37.0)  37 (40.2) | 13 (13.0)  39 (39.0)  48 (48.0) | 4 (12.9)  14 (45.2)  13 (41.9) | <0.001/0.48 |
| **ORIGIN** (n, %)  Fallopian tube  Ovary  Paraovarian  Uncertain | 23 (3.7)  544 (87.5)  17 (2.7)  38 (6.1) | 15 (4.1)  323 (87.8)  15 (4.1)  15 (4.1) | 8 (3.1)  221 (87.0)  2 (0.8)  23 (9.1) | 0  27 (87.1)  2 (6.5)  2 (6.5) | 4 (4.3)  78 (84.8)  0  10 (10.9) | 3 (3.0)  91 (91.0)  0  6 (6.0) | 1 (3.2)  25 (80.6)  0  5 (16.1) | 0.006/0.02 |
| **SUBJECTIVE ASSESSMENT** (n, %)  Benign  Borderline  Malignant | 326 (52.4)  61 (9.8)  235 (37.8) | 316 (85.9)  28 (7.6)  24 (6.5) | 10 (3.9)  33 (13.0)  211 (83.1) | 5 (16.1)  20 (64.5)  6 (19.4) | 3 (3.3)  9 (9.8)  80 (87.0) | 0  4 (4.0)  96 (96.0) | 2 (6.5)  0  29 (93.5) | <0.001/<0.001 |
| **CERTAINTY of subjective assessment** (n, %)  Certainly benign  Probably benign  Uncertain  Probably malignant  Certainly malignant | 176 (28.3)  139 (22.3)  194 (31.2)  80 (12.9)  33 (5.3) | 173 (47.0)  135 (36.7)  24 (6.5)  27 (7.3)  9 (2.4) | 3 (1.2)  4 (1.6)  9 (3.5)  53 (20.9)  185 (72.8) | 2 (6.5)  2 (6.5)  4 (12.9)  16 (51.6)  7 (22.6) | 1 (1.1)  1 (1.1)  4 (4.3)  15 (16.3)  71 (77.2) | 0  0  0  17 (17.0)  83 (83.0) | 0  1 (3.2)  1 (3.2)  5 (16.1)  24 (77.4) | <0.001/<0.001 |
| **ADNEX - Max diameter of lesion**, **mm**(median, IQR) | 65  (44-99) | 53  (39-75) | 88  (60-134) | 52  (41-110) | 112  (74-151) | 85  (58-121) | 88  (66-122) | <0.001/<0.001 |
| **ADNEX - Max diameter of solid component, mm**  (median, IQR) | 18  (0-59) | 0  (0-16) | 60  (37-87) | 14  (6-30) | 63  (43-93) | 64  (42-89) | 73  (46-100) | <0.001/<0.001 |
| **ADNEX - more than 10 cyst locules** (n, %)  No  Yes | 539 (86.7)  83 (13.3) | 338 (91.8)  30 (8.2) | 201 (79.1)  53 (20.9) | 25 (80.6)  6 (19.4) | 70 (76.1)  22 (23.9) | 82 (82.0)  18 (18.0) | 24 (77.4)  7 (22.6) | <0.001/0.77 |
| **ADNEX - Number of papillary projections** (n, %)  0  1  2  3  4 | 484 (77.8)  60 (9.6)  22 (3.5)  14 (2.3)  42 (6.8) | 301 (81.8)  44 (12.0)  12 (3.3)  5 (1.4)  6 (1.6) | 183 (72.0)  16 (6.3)  10 (3.9)  9 (3.5)  36 (14.2) | 12 (38.7)  4 (12.9)  6 (19.4)  4 (12.9)  5 (16.1) | 65 (70.7)  5 (5.4)  3 (3.3)  3 (3.3)  16 (17.4) | 82 (82.0)  4 (4.0)  1 (1.0)  2 (2.0)  11 (11.0) | 24 (77.4)  3 (9.7)  0  0  4 (12.9) | <0.001/<0.001 |
| **ADNEX - Acoustic shadows** (n, %)  No  Yes | 487 (78.3)  135 (21.7) | 259 (70.4)  109 (29.6) | 228 (89.8)  26 (10.2) | 27 (87.1)  4 (12.9) | 83 (90.2)  9 (9.8) | 90 (90.0)  10 (10.0) | 28 (90.3)  3 (9.7) | <0.001/0.96 |
| **ADNEX - Presence of ascites** (n, %)  No  Yes | 558 (89.7)  64 (10.3) | 367 (99.7)  1 (0.3) | 191 (75.2)  63 (24.8) | 30 (96.8)  1 (3.2) | 82 (89.1)  10 (10.9) | 58 (58.0)  42 (42.0) | 21 (67.7)  10 (32.3) | <0.001/<0.001 |
| **COLOR SCORE** (n, %)  1  2  3  4 | 232 (37.3)  168 (27.0)  130 (20.9)  92 (14.8) | 224 (60.9)  110 (29.9)  27 (7.3)  7 (1.9) | 8 (3.1)  58 (22.8)  103 (40.6)  85 (33.5) | 6 (19.4)  13 (41.9)  11 (35.5)  1 (3.2) | 2 (2.2)  19 (20.7)  44 (47.8)  27 (29.3) | 0  18 (18.0)  35 (35.0)  47 (47.0) | 0  8 (25.8)  13 (41.9)  10 (32.3) | <0.001/<0.001 |
| **CYST CONTENT** (n, %)  Anechoic  Ground glass  Hemorragic  Low level  Mixed  Not applicable | 144 (23.2)  63 (10.1)  5 (0.8)  170 (27.3)  65 (10.5)  175 (28.1) | 110 (29.9)  52 (14.1)  4 (1.1)  86 (23.4)  60 (16.3)  56 (15.2) | 34 (13.4)  11 (4.3)  1 (0.4)  84 (33.1)  5 (2.0)  119 (46.9) | 11 (35.5)  3 (9.7)  0  12 (38.7)  2 (6.5)  3 (9.7) | 9 (9.8)  2 (2.2)  1 (1.1)  42 (45.7)  2 (2.2)  36 (39.1) | 12 (12.0)  5 (5.0)  0  23 (23.0)  0  60 (60.0) | 2 (6.5)  1 (3.2)  0  7 (22.6)  1 (3.2)  20 (64.5) | <0.001/<0.001 |
| **KNOWN CA125 at inclusion** (n, %)  No  Yes | 162 (26.0)  460 (74.0) | 118 (32.1)  250 (67.9) | 44 (17.3)  210 (82.7) | 7 (22.6)  24 (77.4) | 15 (16.3)  77 (83.7) | 16 (16.0)  84 (84.0) | 6 (19.4)  25 (80.6) | <0.001/0.83 |
| **CA125** (n, %)  (median IQR) | 23 (11-136) | 13  (10-26) | 126  (27-528) | 20  (10-59) | 120  (22-353) | 322  (106-974) | 42  (17-150) | <0.001/<0.001 |

IQR, interquartile range; FIGO, International Federation of Gynecology and Obstetrics; ADNEX, Assessment of Different NEoplasias in the adnexa; CA, Cancer Antigen. * P-value was calculated with two-sided Pearson’s Chi-Square or Fisher’s Exact or Mann-Whitney or Kruskall-Wallis test as appropriate. The statistical level of significance was set at P<0.05. Of the two p-values reported, the first represents the statistical significance of the differences between benign and malignant tumors, whereas the second represents the statistical significance of the differences between the subtypes of ovarian malignancies (borderline, early stage, advanced and metastatic).

**Table S7.** Histological outcomes of patients in training and validations sets.

| **Histology** | **All tumors** | | | **Benign tumor** | | **Malignant** | | | | | | | |
| --- | --- | --- | --- | --- | --- | --- | --- | --- | --- | --- | --- | --- | --- |
|  |  |  |  |  |  | Borderline | | Early stage | | Advanced | | Metastatic | |
|  | Total  (n= 2,073) | Train  (n= 1,451) | Validation  (n= 622) | Train  (n= 902) | Validation  (n= 368) | Train  (n=82) | Validation  (n=31) | Train  (n= 176) | Validation  (n=92) | Train  (n= 225) | Validation  (n= 100) | Train  (n=66) | Validation  (n=31) |
| **Benign** | 1,270 (61.3) | 902 (62.2) | 368 (59.2) | 902 (100) | 368 (100) | – | – | – | – | – | – | – | – |
| Cystadenoma serous | 203 (16.0) | 151 (10.4) | 52 (8.4) | 151 (16.7) | 52 (14.1) | – | – | – | – | – | – | – | – |
| Cystadenoma mucinous | 94 (7.4) | 64 (4.4) | 30 (4.8) | 64 (7.1) | 30 (8.2) | – | – | – | – | – | – | – | – |
| Cystadenoma sero-mucinous | 5 (0.4) | 2 (0.1) | 3 (0.5) | 2 (0.2) | 3 (0.8) | – | – | – | – | – | – | – | – |
| Cystadenofibroma serous | 185 (14.6) | 132 (9.1) | 53 (8.5) | 132 (14.6) | 53 (14.4) | – | – | – | – | – | – | – | – |
| Cystadenofibroma_mucinous | 13 (1.0) | 9 (0.6) | 4 (0.6) | 9 (1.0) | 4 (1.1) | – | – | – | – | – | – | – | – |
| Cystadenofibroma sero-mucinous | 2 (0.2) | 1 (0.1) | 2 (0.3) | 1 (0.1) | 2 (0.5) | – | – | – | – | – | – | – | – |
| Endometrioma | 196 (15.4) | 126 (8.7) | 70 (11.3) | 126 (14.0) | 70 (19.0) | – | – | – | – | – | – | – | – |
| Teratoma | 247 (19.4) | 184 (12.7) | 63 (10.1) | 184 (20.4) | 63 (17.1) | – | – | – | – | – | – | – | – |
| Struma ovarii | 13 (1.0) | 10 (0.7) | 3 (0.5) | 10 (1.1) | 3 (0.8) | – | – | – | – | – | – | – | – |
| Fibroma/fibrothecoma | 135 (10.6) | 98 (6.8) | 37 (6) | 98 (10.9) | 37 (10.1) | – | – | – | – | – | – | – | – |
| Brenner | 17 (1.3) | 14 (1) | 3 (0.5) | 14 (1.6) | 3 (0.8) | – | – | – | – | – | – | – | – |
| Sactosalpinx | 16 (1.3) | 8 (0.6) | 8 (1.3) | 8 (0.9) | 8 (2.2) | – | – | – | – | – | – | – | – |
| Paraovarian/parasalpingeal cyst | 18 (1.4) | 10 (0.7) | 8 (1.3) | 10 (1.1) | 8 (2.2) | – | – | – | – | – | – | – | – |
| Peritoneal cyst | 8 (0.6) | 6 (0.4) | 2 (0.3) | 6 (0.7) | 2 (0.5) | – | – | – | – | – | – | – | – |
| Simple cyst | 48 (3.8) | 33 (2.3) | 15 (2.4) | 33 (3.7) | 15 (4.1) | – | – | – | – | – | – | – | – |
| Haemorragic corpus luteum | 26 (2.0) | 21 (1.5) | 5 (0.8) | 21 (2.3) | 5 (1.4) | – | – | – | – | – | – | – | – |
| Other | 44 (3.5) | 33 (2.3) | 11 (1.3) | 33 (3.7) | 11 (3.0) | – | – | – | – | – | – | – | – |
| **Borderline** | 113 (5.5) | 82 (5.6) | 31 (5.0) | – | – | 82 (100) | 31 (100) | – | – | – | – | – | – |
| Borderline serous | 66 (58.4) | 46 (3.2) | 20 (3.2) | – | – | 46 (56.1) | 20 (64.5) | – | – | – | – | – | – |
| Borderline mucinous | 46 (40.7) | 35 (2.4) | 11 (1.8) | – | – | 35 (42.7) | 11 (35.5) | – | – | – | – | – | – |
| Borderline Brenner | 1 (0.9) | 1 (0.1) | 0 | – | – | 1 (1.2) | 0 | – | – | – | – | – | – |
| **Early stage**  (FIGO I-II) | 268 (12.9) | 176 (12.1) | 92 (14.8) | – | – | – | – | 176 (100) | 92 (100) | – | – | – | – |
| Serous | 94 (35.1) | 63 (4.3) | 31 (5.0) | – | – | – | – | 63 (35.8) | 31 (33.7) | – | – | – | – |
| Mucinous | 24 (9.0) | 13 (0.9) | 11 (1.8) | – | – | – | – | 13 (7.4) | 11 (12.0) | – | – | – | – |
| Endometrioid | 72 (26.9) | 43 (3.0) | 29 (4.7) | – | – | – | – | 43 (24.4) | 29 (31.5) | – | – | – | – |
| Clear cell | 37 (13.8) | 27 (1.9) | 10 (1.6) | – | – | – | – | 27 (15.3) | 10 (10.9) | – | – | – | – |
| Carcinosarcoma | 2 (0.7) | 1 (0.1) | 1 (0.2) | – | – | – | – | 1 (0.6) | 1 (1.1) | – | – | – | – |
| Granulosa adult | 20 (7.5) | 14 (1.0) | 6 (1.0) | – | – | – | – | 14 (8.0) | 6 (6.5) | – | – | – | – |
| Sertoli-Leydig | 1 (0.4) | 1 (0.1) | 0 | – | – | – | – | 1 (0.6) | 0 | – | – | – | – |
| Immature teratoma | 5 (1.9) | 4 (0.3) | 1 (0.2) | – | – | – | – | 4 (2.3) | 1 (1.1) | – | – | – | – |
| Yolk sac | 1 (0.4) | 0 | 1 (0.2) | – | – | – | – | 0 | 1 (1.1) | – | – | – | – |
| Choriocarcinoma | 1 (0.4) | 1 (0.1) | 0 | – | – | – | – | 1 (0.6) | 0 | – | – | – | – |
| Mixed epithelial | 5 (1.9) | 4 (0.3) | 1 (0.2) | – | – | – | – | 4 (2.3) | 1 (1.1) | – | – | – | – |
| Mixed germinal | 1 (0.4) | 1 (0.1) | 0 | – | – | – | – | 1 (0.6) | 0 | – | – | – | – |
| Other | 5 (1.9) | 4 (0.3) | 1 (0.2) | – | – | – | – | 4 (2.3) | 1 (1.1) | – | – | – | – |
| **Advanced**  **(**FIGO III-IV) | 325 (15.7) | 225 (15.5) | 100 (16.1) | – | – | – | – | – | – | 225 (100) | 100 (100) | – | – |
| Serous | 283 (87.1) | 194 (13.4) | 89 (14.3) | – | – | – | – | – | – | 194 (86.2) | 89 (89.0) | – | – |
| Mucinous | 1 (0.3) | 1 (0.1) | 0 | – | – | – | – | – | – | 1 (0.4) | 0 | – | – |
| Endometrioid | 11 (3.4) | 6 (0.4) | 5 (0.8) | – | – | – | – | – | – | 6 (2.7) | 5 (5.0) | – | – |
| Clear cell | 6 (1.8) | 6 (0.4) | 0 | – | – | – | – | – | – | 6 (2.7) | 0 | – | – |
| Carcinosarcoma | 7 (2.2) | 4 (0.3) | 3 (0.5) | – | – | – | – | – | – | 4 (1.8) | 3 (3.0) | – | – |
| Granulosa adult | 1 (0.3) | 0 | 1 (0.2) | – | – | – | – | – | – | 0 | 1 (1.0) | – | – |
| Sertoli-Leydig | 1 (0.3) | 1 (0.1) | 0 | – | – | – | – | – | – | 1 (0.4) | 0 | – | – |
| Immature teratoma | 1 (0.3) | 1 (0.1) | 0 | – | – | – | – | – | – | 1 (0.4) | 0 | – | – |
| Yolk sac | 1 (0.3) | 1 (0.1) | 0 | – | – | – | – | – | – | 1 (0.4) | 0 | – | – |
| Mullerian carcinoma | 6 (1.8) | 5 (0.3) | 1 (0.2) | – | – | – | – | – | – | 5 (2.2) | 1 (1.0) | – | – |
| Mixed epithelial | 2 (0.6) | 2 (0.1) | 0 | – | – | – | – | – | – | 2 (0.9) | 0 | – | – |
| Mixed germinal | 2 (0.6) | 1 (0.1) | 1 (0.2) | – | – | – | – | – | – | 1 (0.4) | 1 (1.0) | – | – |
| Other | 3 (0.9) | 3 (0.2) | 0 | – | – | – | – | – | – | 3 (1.3) | 0 | – | – |
| **Metastatic** | 97 (4.7) | 66 (4.5) | 31 (5.0) | – | – | – | – | – | – | – | – | 66 (100) | 31 (100) |
| Gynecological | 12 (12.4) | 8 (0.6) | 4 (0.6) | – | – | – | – | – | – | – | – | 8 (12.1) | 4 (12.9) |
| Breast | 11 (11.3) | 9 (0.6) | 2 (0.3) | – | – | – | – | – | – | – | – | 9 (13.6) | 2 (6.5) |
| Gastrointestinal | 52 (53.6) | 36 (2.5) | 16 (2.6) | – | – | – | – | – | – | – | – | 36 (54.5) | 16 (51.6) |
| Krukenberg | 7 (7.2) | 5 (0.3) | 2 (0.32) | – | – | – | – | – | – | – | – | 5 (7.6) | 2 (6.5) |
| Other | 15 (15.5) | 8 (0.6) | 7 (1.1) | – | – | – | – | – | – | – | – | 8 (12.1) | 7 (22.6) |

**Table S8.** Distribution of tumor histology and FIGO stages (n=2073).

|  | **Benign**  **(n, %)** | **Malignant** | | | | |
| --- | --- | --- | --- | --- | --- | --- |
|  |  | All malignant  (n, %) | Borderline  (n, %) | Early  (FIGO I-II)  (n, %) | Advanced (FIGO III-IV)  (n, %) | Metastatic  (n, %) |
| TRAINING SET  n= 1451 | 902  (62.16 %) | 549  (37.83 %) | 82  (5.65 %) | 176  (12.13 %) | 225  (15.51 %) | 66  (4.54 %) |
| VALIDATION SET  n= 622 | 368  (59.16 %) | 254  (40.84 %) | 31  (4.98 %) | 92  (14.79 %) | 100  (16.08 %) | 31  (4.99 %) |
| TOTAL  n= 2073 | 1270  (61.26 %) | 803  (38.74 %) | 113  (5.45 %) | 268  (12.93 %) | 325  (15.68 %) | 97  (4.68 %) |

FIGO, International Federation of Gynecology and Obstetrics. Percentages are row percentages

**Table S9.** SHapley Additive exPlanations (SHAP) analysis of feature importance in the radiomics-only model.

| **Feature** | **SHAP_Train** | **SHAP_Test** | **Overfit_Score_Absolute_value** | **Overfit_Score** |
| --- | --- | --- | --- | --- |
| F_rlm.glnu.norm | 0.574113 | 0.516529 | 0.057583213 | 0.057583 |
| F_cm.info.corr.2 | 0.571895 | 0.526334 | 0.045560479 | 0.04556 |
| F_szm.glnu.norm | 0.353004 | 0.3278 | 0.025204003 | 0.025204 |
| F_cm.clust.tend | 0.231096 | 0.212153 | 0.018943712 | 0.018944 |
| F_rlm.glnu | 0.224246 | 0.206597 | 0.017649874 | 0.01765 |
| F_rlm.rl.var | 0.307808 | 0.291874 | 0.01593411 | 0.015934 |
| F_stat.entropy | 0.301057 | 0.287504 | 0.013553113 | 0.013553 |
| F_cm.inv.var | 0.401807 | 0.388882 | 0.012924552 | 0.012925 |
| F_szm.zsnu.norm | 0.393827 | 0.385473 | 0.008354068 | 0.008354 |
| F_szm.glnu | 0.363858 | 0.356415 | 0.007443428 | 0.007443 |
| F_cm.auto.corr | 0.760677 | 0.758477 | 0.002199709 | 0.0022 |
| F_cm.inv.diff.mom.norm | 0.324612 | 0.326274 | 0.001662165 | -0.00166 |
| F_szm.lzhge | 0.295611 | 0.298758 | 0.003147036 | -0.00315 |
| F_stat.kurt | 0.764121 | 0.775985 | 0.011864126 | -0.01186 |

The features contributing most to overfitting in the radiomics-only model were identified using SHapley Additive exPlanations (SHAP) values ^4^. SHAP values quantify each feature’s contribution to model predictions in a consistent and interpretable manner. We compared the mean absolute SHAP value of each feature in the training and test datasets. Features with high SHAP importance in the training set but lower importance in the test set are likely to be overfitted. We defined an “Overfit_Score” as the difference in a feature’s SHAP importance between the training and test set.

The two features with the highest Overfit_Scores were: F_rlm.glnu.norm (Overfit_Score: 0.0576) and F_cm.info.corr.2 (0.0456). Both these features represent heterogenous echogenicity. F_rlm.glnu.norm indicates how similar the lengths of sequences of consecutive identical pixels are; a low value means the sequences are similar and the echogenicity is homogeneous, and vice versa. F_cm.info.corr.2’ is a texture features belonging to the class of gray level co-occurrence matrix (GLCM) features based on information theory. It relates to the randomness in data, quantifying the complexity of the texture. Lower values of the feature (low information) correspond to a more uniform or plain pattern of the texture, typical of benign tumors, while high values of the feature (high information) means that image texture is more complex with more variations, typical of malignant tumors).

| **Feature selection alternative method** | **Selected features**  **(n, specific features)** | **AUC** | | **ACCURACY** | | **SENSITIVITY** | | **SPECIFICITY** | |
| --- | --- | --- | --- | --- | --- | --- | --- | --- | --- |
|  |  | TRAIN | TEST | TRAIN | TEST | TEST | TEST | TRAIN | TEST |
| Univariate analysis | n= 14:  'F_stat.kurt',  'F_stat.entropy',  'F_cm.inv.diff.mom.norm',  'F_cm.inv.var',  'F_cm.auto.corr',  'F_cm.clust.tend',  'F_cm.info.corr.2',  'F_rlm.glnu',  'F_rlm.glnu.norm',  'F_rlm.rl.var',  'F_szm.lzhge',  'F_szm.glnu',  'F_szm.glnu.norm',  'F_szm.zsnu.norm' | 0.90 | 0.81 | 0.83 | 0.75 | 0.83 | 0.73 | 0.82 | 0.77 |
| Recursive Feature Elimination (RFE)  Best cut-off: 0.42 | n= 14:  'F_stat.skew',  'F_stat.rms',  'F_cm.joint.avg',  'F_cm.sum.avg',  'F_cm.sum.entr',  'F_cm.auto.corr',  'F_cm.clust.shade',  'F_cm.info.corr.2',  'F_rlm.srhge',  'F_rlm.rlnu',  'F_rlm.gl.var',  'F_rlm.rl.var',  'F_szm.hgze',  'F_szm.zs.var | 0.96 | 0.78 | 0.88 | 0.71 | 0.91 | 0.68 | 0.86 | 0.73 |
| Minimum Redundancy Maximum Relevance (MRMR)  Best cut-off: 0.43 | n= 14:  'F_stat.entropy',  'F_cm.diff.entr',  'F_stat.max',  'F_cm.info.corr.2',  'F_stat.uniformity',  'F_cm.clust.shade',  'F_stat.skew',  'F_rlm.glnu.norm',  'F_stat.mad',  'F_cm.inv.diff.mom.norm',  'F_cm.joint.max',  'F_stat.rms',  'F_stat.range',  'F_cm.energy' | 0.84 | 0.80 | 0.76 | 0.73 | 0.75 | 0.68 | 0.77 | 0.77 |

**Table S10.** Comparison of radiomics-only model performance with different feature selection methods.

**References**

1 Van Calster B, Valentin L, Froyman W, et al. Validation of models to diagnose ovarian cancer in patients managed surgically or conservatively: multicentre cohort study. *BMJ*. Published online July 30, 2020:m2614. doi:10.1136/bmj.m2614

2. Scapicchio C, Gabelloni M, Barucci A, Cioni D, Saba L, Neri E. A deep look into radiomics. *Radiol Med*. 2021;126(10):1296-1311. doi:10.1007/s11547-021-01389-x

3. Barreñada L, Ledger A, Dhiman P, et al. ADNEX risk prediction model for diagnosis of ovarian cancer: systematic review and meta-analysis of external validation studies. *BMJ Medicine*. 2024;3(1):e000817. doi:10.1136/bmjmed-2023-000817

4. Lundberg S, Lee SI. A Unified Approach to Interpreting Model Predictions. Published online November 25, 2017. https://arxiv.org/abs/1705.07874

**Figure S1.** Principal Component Analysis (PCA) of radiomics features in training set.

The plot shows the first two Principal Components (PC1 and PC2) derived from the radiomics features used in the model. Each point represents an image, and colors indicate the manufacturer of the ultrasound machine. PC1 and PC2 account for 28.5% and 12.3% of the total variance, respectively. The similarity observed along PC1 and PC2 suggests good agreement in the selected features between ultrasound machines produced by different companies.

**
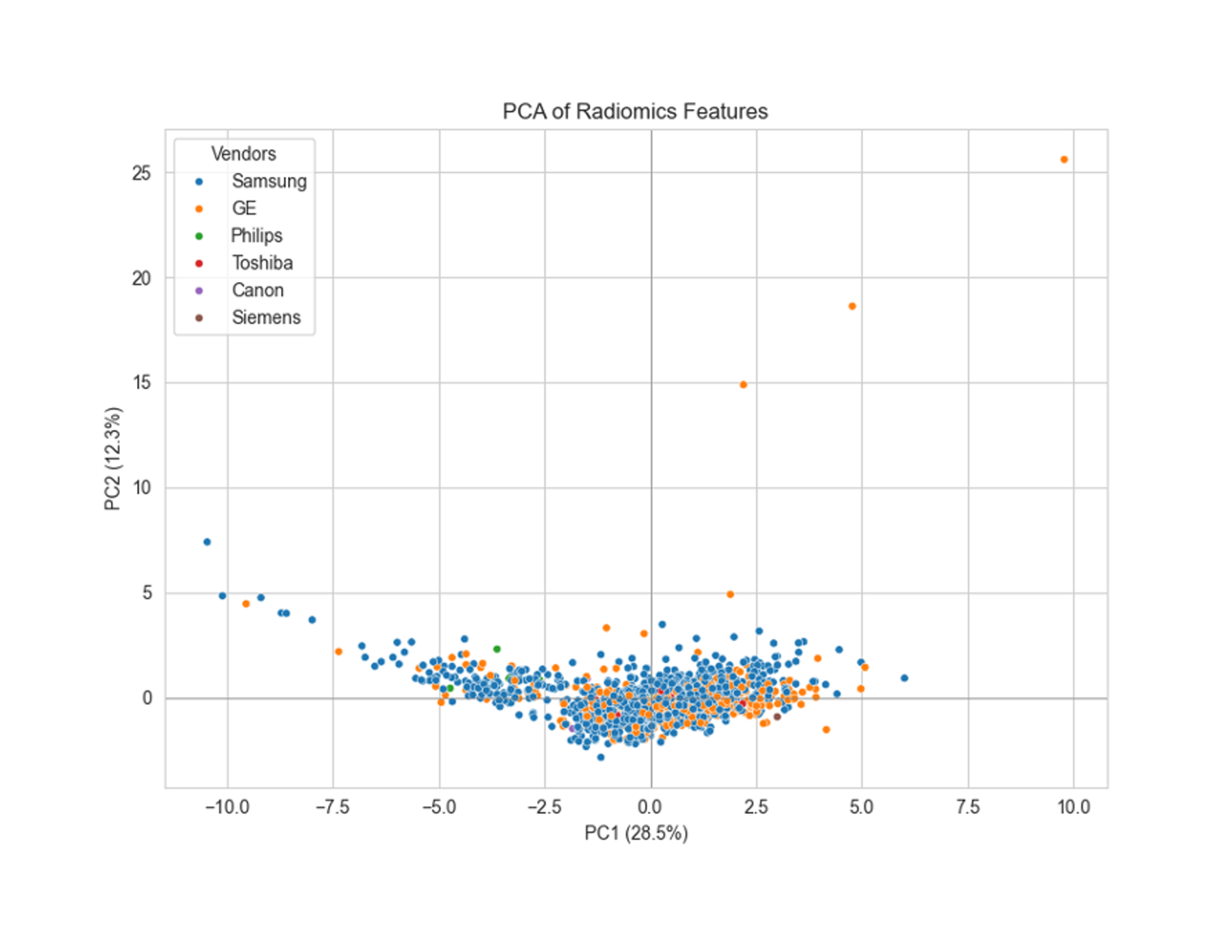
**

**Figure S2.** Ranking of feature importance in (a) clinical-radiomics model and (b) radiomics-only model. This figure displays the relative importance of radiomics and clinical features in the training set. In the clinical-radiomics model, CA125 was the most impactful feature in predicting benign vs malignant masses (importance value 0.19), followed by radiomics statistical feature “F.stat.kurt” (importance value=0.11). In radiomics-only model, radiomics statistical feature “F.stat.kurt” was the most impactful feature in predicting benign vs malignant masses (importance value 0.33), followed by radiomics textural features “F_szm.glnu.norm” and “F_cm.info.corr.2” (importance value 0.083 and 0.076, respectively).


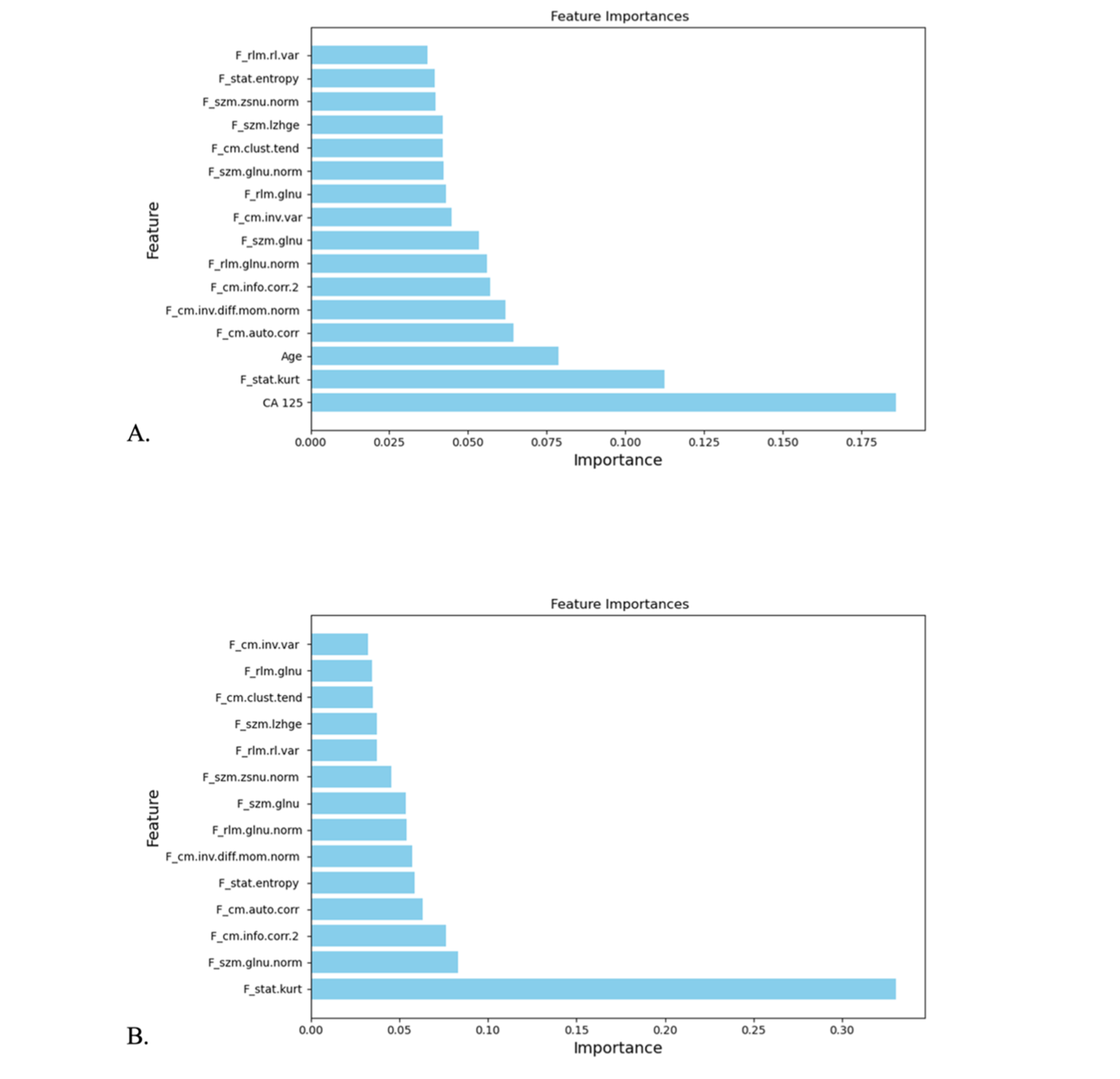


**Figure S3.** Distribution of kurtosis (F.stat.kurt) feature in benign and malignant masses. (A) Violin plot of the “F.stat.kurt” distribution for the 2,073 patients included in the study (outliers removed), including 1,270 benign (0) and 803 malignant (1) tumors. The white line corresponds to the median kurtosis values (76.65 for benign and 32.19 for malignant masses). The black line corresponds to the min to the max values of the feature and the black box indicates the interquartile range (25^th^-75^th^). The blue width of the violin represents the data density (wider area indicates more concentrated value).

(B) Representative ultrasound images of three benign masses (b1, b2, b3) and of three malignant masses (m1, m2, m3). (C) Distribution density plot of pixel intensities calculated within the Region of Interest (ROI) of the six masses. A sharper and more peaked distribution of pixel intensities, reflecting a higher kurtosis, is visible for benign masses, whereas a flatter distribution of pixel intensities, reflecting low kurtosis, is observed in malignant masses which are more heterogeneous.

**
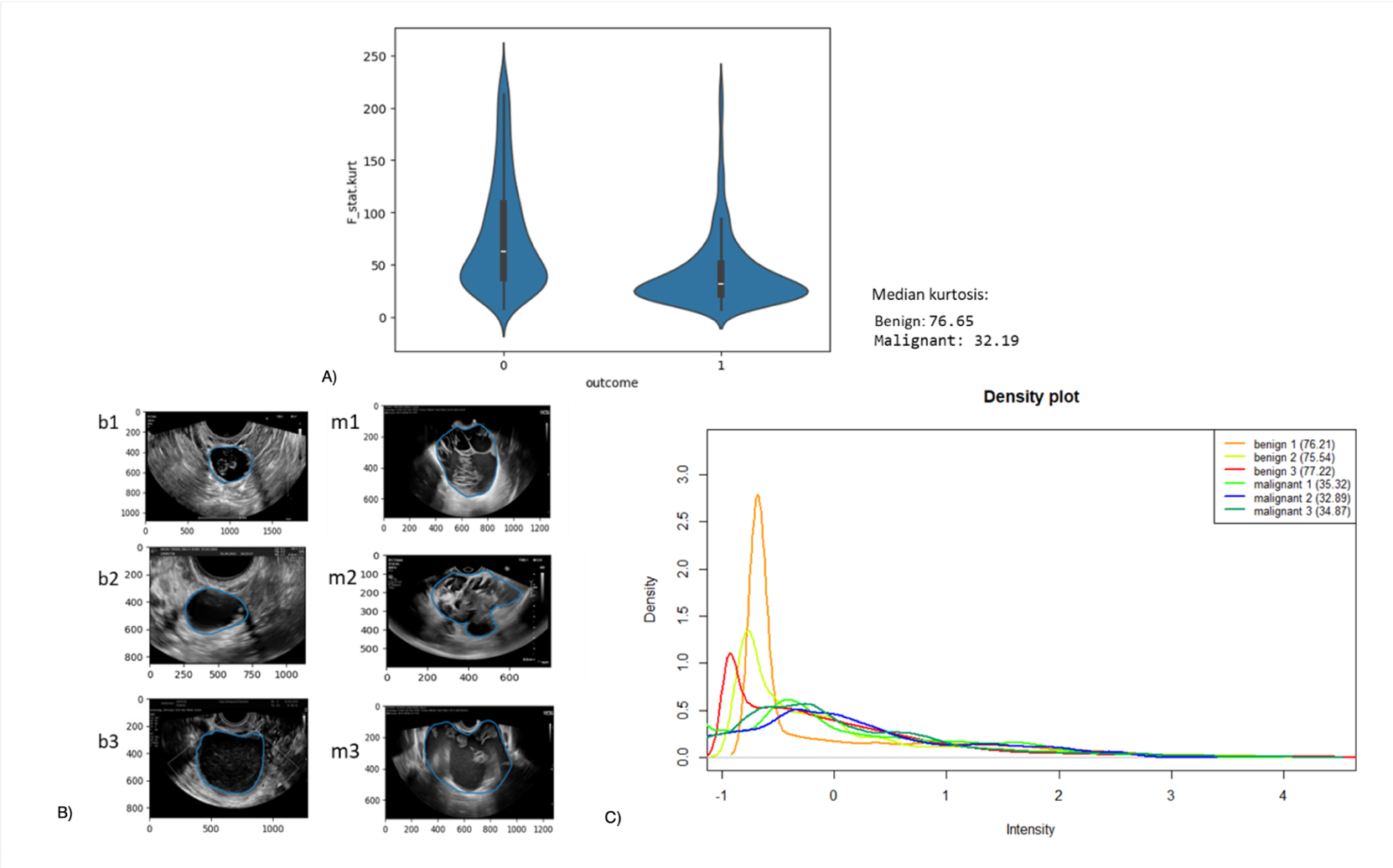
**


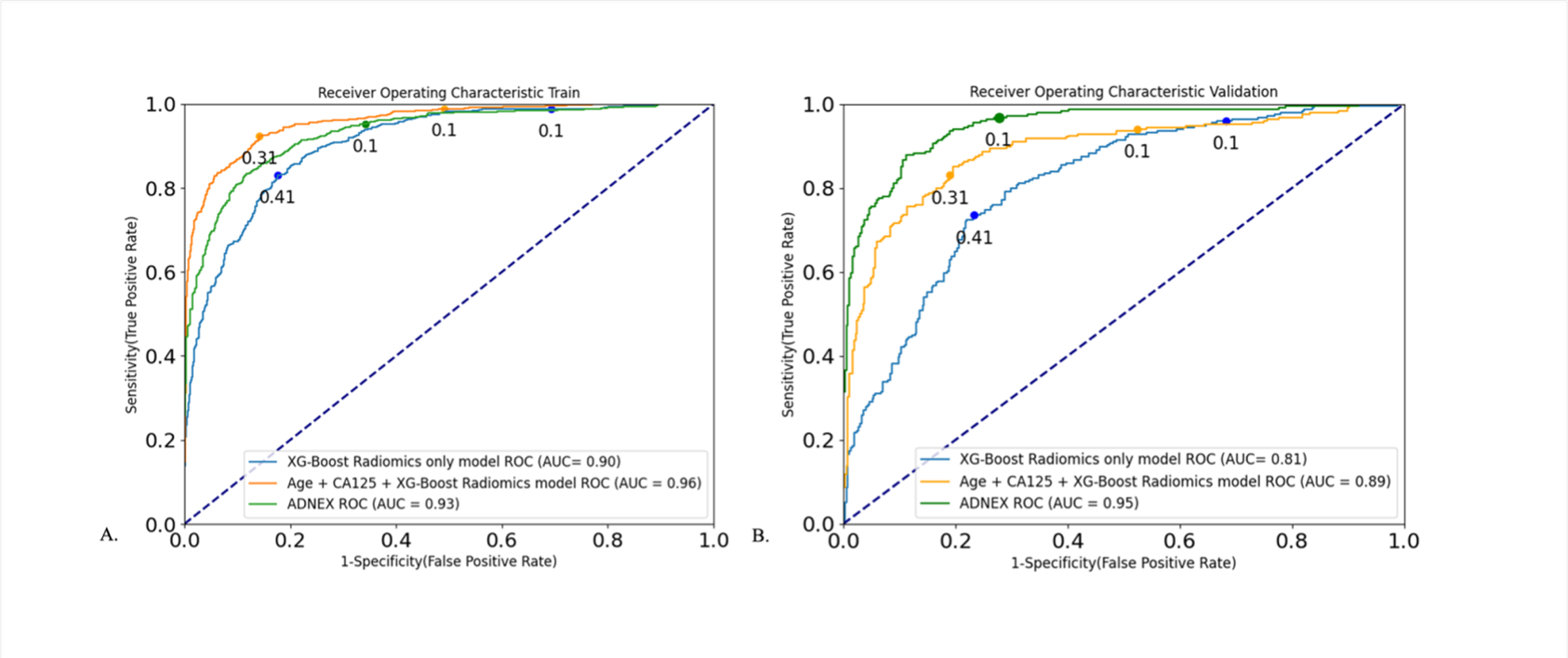
**Figure S4.** Receiver-operating-characteristics (ROC) curves for radiomics-only, clinical-radiomics and ADNEX models in (a) training set (*n* = 1451, of which 549 (37.83%) were malignant) and (b) validation set (*n* = 622, of which 254 (40.84%) were malignant). AUC= area under the ROC curve. The bullets on the ROC curves denote the 10% risk of malignancy cutoff (0.1) and the “best cut-off” for the radiomic-only model and the clinical-radiomics model (age + CA125 + radiomics), the best cutoff being defined as the threshold that maximizes the proportion of correctly classified cases based on Youden’s index (41% and 31%, respectively).
